# Reproductive outcome after frozen embryo transfer with hormone replacement therapy according to luteal‐phase support protocol: systematic review and network meta‐analysis of randomized controlled trials

**DOI:** 10.1002/uog.29302

**Published:** 2025-08-01

**Authors:** A. Etrusco, B. Ata, V. Agrifoglio, A. D'Amato, C. Wyns, A. Vitagliano, C. Alviggi, A. Conforti, A. Favilli, P. De Franciscis, A. S. Laganà, G. Riemma

**Affiliations:** ^1^ Department of Health Promotion, Mother and Child Care, Internal Medicine and Medical Specialties (PROMISE) University of Palermo Palermo Italy; ^2^ Unit of Obstetrics and Gynecology, ‘Paolo Giaccone’ Hospital Palermo Italy; ^3^ Department of Obstetrics and Gynecology Koç University School of Medicine Istanbul Türkiye; ^4^ ART Fertility Clinics Dubai United Arab Emirates; ^5^ Department of Interdisciplinary Medicine (DIM), Unit of Obstetrics and Gynecology University of Bari ‘Aldo Moro’, Policlinico of Bari Bari Italy; ^6^ Cliniques Universitaires Saint‐Luc Université Catholique de Louvain Brussels Belgium; ^7^ Department of Public Health University of Naples Federico II Napoli Italy; ^8^ Department of Neuroscience, Reproductive Science and Odontostomatology University of Naples Federico II Napoli Italy; ^9^ Section of Obstetrics and Gynecology, Department of Medicine and Surgery University of Perugia Perugia Italy; ^10^ Department of Woman, Child and General and Specialized Surgery University of Campania ‘Luigi Vanvitelli’ Naples Italy

**Keywords:** artificial cycle, cumulative pregnancy rate, frozen embryo transfer, hormone replacement therapy, infertility, luteal phase support, progesterone

## Abstract

**Objective:**

To compare reproductive outcome between luteal‐phase support (LPS) protocols for frozen embryo transfer (FET) cycles with hormone replacement therapy (HRT).

**Methods:**

A search was conducted in MEDLINE, Scopus, LILACS, EMBASE, Scielo.br, PROSPERO, CINAHL, PsycINFO, AMED, ClinicalTrials.gov, ICTRP, the Cochrane Library and conference proceedings, with no restrictions on date, geography or language. We included all randomized controlled trials (RCTs) that allocated infertile women to at least two different hormone‐based LPS protocols for HRT–FET, with similar baseline characteristics between groups. The Preferred Reporting Items for Systematic reviews and Meta‐Analyses extension statement for network meta‐analyses (PRISMA‐NMA) was followed. A random‐effects network meta‐analysis was performed for direct and indirect pairwise comparisons to rank available LPS protocols by the surface under the cumulative ranking curve area (SUCRA). Risk of bias was assessed using the Cochrane risk‐of‐bias tool version 1. Certainty of evidence was evaluated using the Confidence in Network Meta‐Analysis (CINeMA) criteria. The primary outcomes were the live birth rate and the combined rate of ongoing pregnancy and live birth; the secondary outcomes were the clinical pregnancy rate and the pregnancy loss rate.

**Results:**

Ten RCTs assigned a total of 4216 patients to nine different LPS approaches. Regarding the combined outcome of ongoing pregnancy and live birth, oral dydrogesterone (DYD) combined with gonadotropin‐releasing hormone agonist (GnRHa) was significantly more efficacious compared with all other LPS protocols (very low to low certainty of evidence), with SUCRA analysis ranking it as the treatment of choice (SUCRA = 97.3%). When the analysis was restricted to live birth only, vaginal suppository progesterone showed a higher likelihood of being the treatment of choice (SUCRA = 89.7%), but only exhibited a significant difference on pairwise analysis when compared with intramuscular progesterone (odds ratio (OR), 0.53 (95% CI, 0.33–0.84); low certainty of evidence) and intramuscular progesterone + vaginal suppository progesterone (OR, 0.47 (95% CI, 0.32–0.69); low certainty of evidence). For the clinical pregnancy rate, no significant differences between treatments were found (very low to low certainty of evidence), with vaginal suppository progesterone + human chorionic gonadotropin being the highest‐ranked treatment (SUCRA = 33.7%). For pregnancy loss rate, intramuscular progesterone + vaginal suppository progesterone was significantly more efficacious compared with either treatment alone (low certainty of evidence), and had the highest chance of being the top‐ranked treatment (SUCRA = 51.4%).

**Conclusions:**

There is very‐low‐to‐low‐certainty evidence that oral DYD + GnRHa and vaginal suppository progesterone alone could be the most promising LPS approaches to increase the rates of live birth and ongoing pregnancy in women undergoing HRT–FET. However, the low certainty of evidence and the lack of a clear first‐ranked treatment, due to inconsistencies in the analysis for some outcomes, stress the need for further RCTs on this subject. © 2025 The Author(s). *Ultrasound in Obstetrics & Gynecology* published by John Wiley & Sons Ltd on behalf of International Society of Ultrasound in Obstetrics and Gynecology.

## INTRODUCTION

Approximately 40 years since the first frozen embryo transfer (FET), almost 40% of all *in‐vitro* fertilization cycles in Europe now involve FET^1^, ^2^. This shift has been driven by advances in vitrification technology^3^, ^4^, which have significantly increased embryo cryosurvival rates and improved FET outcomes^5^, ^6^. Other factors contributing to the increased use of FET cycles include a reduced risk of ovarian hyperstimulation syndrome compared with fresh embryo transfer cycles, as well as the growing use of preimplantation genetic testing and single embryo transfer^7^, ^8^, ^9^.

FET can be performed in a natural menstrual cycle, a modified–natural cycle or a hormone replacement therapy (HRT) cycle, which is also known as an artificial cycle and involves the sequential administration of estrogen and progesterone^10^, ^11^, ^12^. HRT–FET has been used widely because of the flexibility it offers to both the patient and the physician, and its lack of dependence on a menstrual cycle. Debate over the optimal method of endometrial preparation is ongoing^13^; however, it appears that HRT cycles may be associated with a higher risk of pregnancy complications, such as early pregnancy loss, gestational hypertension, pre‐eclampsia, postpartum hemorrhage and preterm delivery^14^. Nevertheless, HRT cycles are still considered safe for patients at low risk for these complications and are performed widely^15^. The secretory transformation of the endometrium and maintenance of a pregnancy depend on adequate supplementation of exogenous progesterone in a HRT–FET cycle^16^.

In a HRT–FET cycle, progesterone can be administered via an oral, intramuscular (IM), vaginal or subcutaneous (SC) route. Oral administration of progesterone is both comfortable and convenient; however, adequate secretory transformation of the endometrium is not always achieved using this route because of the low bioavailability of oral progesterone after the hepatic first‐pass effect^17^. A recent trial suggests that oral dydrogesterone (DYD), a synthetic progesterone, has similar efficacy and safety to vaginal progesterone gel^18^. On the other hand, IM and SC progesterone administration are commonly used because of their ability to achieve higher serum progesterone levels and satisfactory rates of clinical pregnancy^19^. However, IM progesterone is often poorly tolerated because of painful daily injections and inflammation at the injection site^20^. The vaginal route maintains a stable progesterone concentration in the endometrium, even with low serum progesterone levels, reducing the risk of systemic side effects^21^. However, some patients find vaginal progesterone uncomfortable, and others may have serum progesterone levels that are too low to support implantation and successful pregnancy^16^, ^17^. Protocols involving the addition of human chorionic gonadotropin (hCG) or gonadotropin‐releasing hormone agonist (GnRHa) to exogenous progesterone^22^, ^23^, as well as those combining multiple routes of progesterone administration^24^, have been trialed, with variable results. Ultimately, the optimal protocol for luteal‐phase support (LPS) in HRT–FET cycles remains to be defined^25^.

The purpose of this systematic review and network meta‐analysis was to compare reproductive outcomes following HRT–FET between several LPS protocols available in the literature.

## METHODS

This systematic review and network meta‐analysis was conducted in accordance with the methodological specifications outlined by Mbuagbaw *et al*.^26^ and the Cochrane Handbook for Systematic Reviews of Interventions^27^. The extension statement of the Preferred Reporting Items for Systematic reviews and Meta‐Analyses dedicated to network meta‐analyses (PRISMA‐NMA)^28^ was followed. On 7 May 2024, the study protocol was registered in the International Prospective Register of Systematic Reviews (PROSPERO) database (registration number: CRD42024540496).

### Data sources and search strategy

The electronic databases MEDLINE (accessed through PubMed), Scopus, LILACS, EMBASE, Scielo.br and PROSPERO were searched using the following medical subject heading (MeSH) terms and keywords: ‘luteal phase support’ AND ‘hormone replacement therapy’ (MeSH unique ID: D020249) OR ‘artificial cycle’ AND ‘frozen‐thawed embryo transfer’ OR ‘frozen embryo transfer’, without any date, geographic or language restrictions. The search string was modified according to the syntax of each database (Appendix S1).

In order to minimize publication bias, additional searches were carried out to locate other pertinent papers on CINAHL, PsycINFO and AMED. To identify further randomized controlled trials (RCTs), we also searched ClinicalTrials.gov, the Cochrane Central Register of Controlled Trials (CENTRAL) and the International Clinical Trials Registry Platform (ICTRP) of the World Health Organization. In addition, a search for national and international conference abstracts was conducted by examining the gray literature (NTIS, PsycEXTRA). Included studies and relevant reviews were searched manually for additional citations.

### Study selection

We included RCTs that allocated infertile women to at least two different hormone‐based LPS protocols (involving different treatments and/or routes of administration) for HRT–FET, with similar baseline characteristics between the groups. We excluded studies that evaluated non‐hormonal interventions; those in which treatment protocols differed only by dose or were indistinguishable between groups; and those that evaluated natural‐cycle ET or a different ovulation induction protocol not involving HRT–FET.

### Data extraction

Data extraction forms were created specifically for this network meta‐analysis. The following characteristics were extracted: patient clinical description, study duration and setting, inclusion and exclusion criteria, characteristics of assisted reproductive technology protocol, treatment approaches, reproductive outcomes, duration of treatment and follow up, and quality elements.

Two authors (A.E., G.R.) independently reviewed and categorized each abstract as eligible or ineligible for full‐text review. Any disagreement was resolved by discussion with a third author (P.D.F.). The same two authors examined the full text of the selected studies and separately extracted relevant information on study characteristics and findings to determine potential applicability. Disagreement was resolved by discussion with three other authors (A.D., A.V., A.S.L.) until consensus was reached. When the study procedures indicated that additional outcome data were gathered, unpublished data were obtained by contacting the original study authors directly.

### Assessment of risk of bias

The Cochrane risk‐of‐bias tool version 1 (RoB 1)^29^ was used to evaluate the risk of bias of the included RCTs. The following seven domains were critically appraised: random sequence generation; allocation concealment; blinding of participants and personnel; blinding of outcome assessment; incomplete outcome data; selective reporting; and other bias. Detailed methodology is provided in Table S1. The risk of bias was rated independently by three authors (A.C., A.F., C.W.) as low, high or unclear. Disagreement was resolved after concealment, by discussion with three other authors (A.V., C.A., V.A.).

To further measure the trustworthiness of included RCTs, we evaluated the following: whether the RCT had been preregistered in a publicly available international trial registry before randomization (for RCTs that began fewer than 15 years ago); approval by an ethics committee or an institutional review board; adherence to the CONsolidated Standards of Reporting Trials (CONSORT) statement; feasibility and comparability of both patient characteristics at baseline and reported outcomes (e.g. studies reporting live birth rate (LBR) fewer than 9 months after randomization were excluded); and consistency of the primary outcome and sample size between the published manuscript and the version registered in an online RCT database. Additionally, we also checked for retractions or expressions of concern related to eligible studies and their authors on PubMed and Retraction Watch.

The certainty of evidence was assessed using the Confidence in Network Meta‐Analysis (CINeMA) criteria^30^.

### Outcomes

The primary outcome of this network meta‐analysis was LBR, defined as the birth of a living fetus ≥ 22 weeks' gestation. For studies that did not report the LBR, we used a surrogate primary outcome of the ongoing pregnancy rate (OPR), defined as ongoing pregnancy ≥ 22 weeks' gestation. Hence, we defined the surrogate outcome OPR/LBR as the total rate of live births and ongoing pregnancies ≥ 22 weeks' gestation. The secondary outcomes were clinical pregnancy rate (CPR), defined as ultrasonographic visualization of one or more intrauterine gestational sacs, and pregnancy loss rate (PLR), defined as a non‐viable intrauterine pregnancy with either an empty gestational sac or a gestational sac containing an embryo or fetus without fetal heart activity within the first 12 + 6 weeks of gestation.

### Data synthesis

We used STATA version 14.1 (StataCorp, College Station, TX, USA) to analyze data and produce graphs. To check if our results were consistent, we used the command ‘<*network meta consistency*>’, which tests whether the assumption of global consistency is statistically verified for each significant result. Next, we used the separating indirect from direct evidence (SIDE)‐splitting method^27^, using the command ‘<*network sidesplit all*>’, as a local approach to identify the presence of discrepancies between direct and indirect evidence when comparing LPS protocols. The consistency assumption was considered valid when no significant differences between direct and indirect evidence for pairwise comparisons were found by global and local testing. In this instance, the consistency model indicated that any observed outcome variation was the result of random errors and/or the intervention's true effect. This suggests that both direct and indirect comparisons would produce relevant results.

We analyzed data using the DerSimonian–Laird random‐effects model, which accounts for variability between studies when estimating overall effect sizes. The results were presented as odds ratios (ORs) with 95% CI. We assessed between‐study heterogeneity using the Higgins *I* ^2^ score, which quantifies the percentage of total variation across the studies that is due to heterogeneity rather than chance, with thresholds of > 25%, > 50% and > 75% considered to represent low, moderate and high heterogeneity, respectively. Sensitivity analyses were carried out when > 50% heterogeneity was present in order to identify pertinent causes of heterogeneity by restricting the analysis to subsets of studies with inherent similarities. For example, we conducted analyses in which we excluded studies with a mean maternal age over 35 years, those that used cleavage‐stage embryo transfer and those that did not report endometrial thickness at the time of embryo transfer.

We used funnel plots to check for publication bias. For example, if studies appear unevenly distributed on one side of the plot, it could suggest missing unpublished studies with negative or non‐significant results, and if smaller studies tend to show exaggerated effects, it may indicate selective reporting. This visual analysis was complemented by Egger's test. Finally, for each outcome under investigation, a ranking plot was produced using the surface under the cumulative ranking curve area (SUCRA) method and a league table of ORs for direct and indirect comparisons was created, in order to assess the relative effectiveness of the various LPS protocols.

## RESULTS

### Study selection

**Figure 1:**
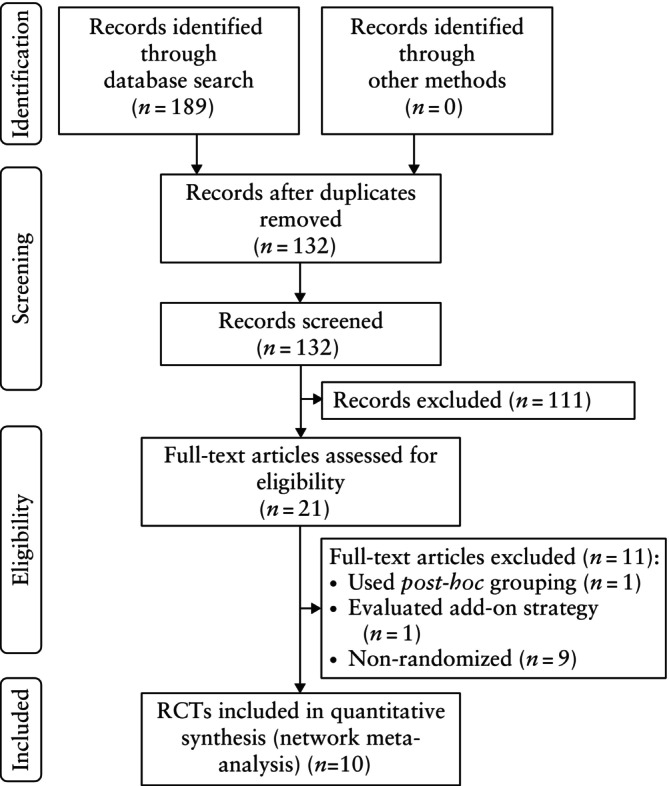
PRISMA flowchart summarizing inclusion of studies in systematic review and network meta‐analysis. RCT, randomized controlled trial.

One hundred and eighty‐nine studies were identified initially via the database search (Figure 1). Of those, 57 were removed as duplicates, and a further 111 records were excluded following title and abstract screening. Twenty‐one studies underwent full‐text assessment, of which one was excluded for evaluating mixed‐route treatment protocols using *post‐hoc* grouping, and another was excluded for evaluating the effect of an add‐on strategy (i.e. an additional dose) in two retrospective cohorts (Appendix S2). Of the remaining studies^22^, ^23^, ^31^, ^32^, ^33^, ^34^, ^35^, ^36^, ^37^, ^38^, ^39^, ^40^, ^41^, ^42^, ^43^, ^44^, ^45^, ^46^, ^47^, nine were excluded for being non‐randomized, leaving 10 RCTs that were included in the network meta‐analysis.

### Study characteristics

The main characteristics of the 10 RCTs^22^, ^33^, ^34^, ^35^, ^36^, ^37^, ^40^, ^41^, ^44^, ^47^ included in the quantitative synthesis are summarized in Table S2. One study was from Türkiye^36^, two were from China^22^, ^44^, one was from the USA^33^, two were from Japan^40^, ^41^, two were from Iran^37^, ^47^ and two were from Israel^34^, ^35^. A total of 4216 patients were included. The following LPS protocols were assessed by the RCTs: IM progesterone^22^, ^33^, ^34^, ^35^, ^36^, ^37^, ^44^, vaginal gel progesterone^35^, ^36^, ^37^, ^40^, ^44^, ^47^, vaginal suppositories of micronized progesterone (henceforth referred to as vaginal suppository progesterone)^33^, ^34^, ^35^, ^37^, ^40^, ^41^, ^47^, IM progesterone + vaginal suppository progesterone^33^, oral DYD^36^, ^37^, ^47^, oral DYD + GnRHa^47^, oral DYD + hCG^47^, vaginal suppository progesterone + hCG^41^ and IM progesterone + hCG^22^.

### Risk of bias of included studies

Methodological quality assessment is summarized and illustrated in Table S1 and Figure S1, respectively. Overall, three studies were deemed to be at low risk of bias^22^, ^33^, ^37^, while five studies were considered at unclear risk^35^, ^36^, ^40^, ^44^, ^47^ and two studies were considered at high risk^34^, ^41^. In detail, as most RCTs had an open‐label design, most were at high risk for performance bias because of the absence of blinding of participants or personnel^22^, ^34^, ^36^, ^40^, ^41^, ^44^, ^47^. Similarly, outcome assessment was unblinded in seven studies^34^, ^36^, ^37^, ^40^, ^41^, ^44^, ^47^, rendering them at high risk for detection bias. Conversely, the majority of RCTs were at low risk of bias for random sequence generation^22^, ^33^, ^34^, ^36^, ^40^, ^44^, ^47^ and unclear risk of bias for allocation concealment^22^, ^35^, ^36^, ^40^, ^44^, ^47^ and other bias^22^, ^35^, ^36^, ^37^, ^40^, ^41^, ^47^. Moreover, all trials were at low risk for attrition and reporting biases. Eight RCTs^22^, ^33^, ^34^, ^36^, ^37^, ^40^, ^44^, ^47^ were registered in a valid prospective repository before participant enrollment, while registration details were not available for the other two RCTs^35^, ^41^ because their enrollment started before 2010, when trial registration was not universally mandatory. According to funnel plot analysis (Figure S2) and Egger's test (*P* = 0.408), no publication bias was detected for the outcome of CPR. Publication bias was not assessed for the other outcomes as they were reported in fewer than 10 studies.

### Synthesis of results

#### Ongoing pregnancy rate and live birth rate

**Figure 2:**
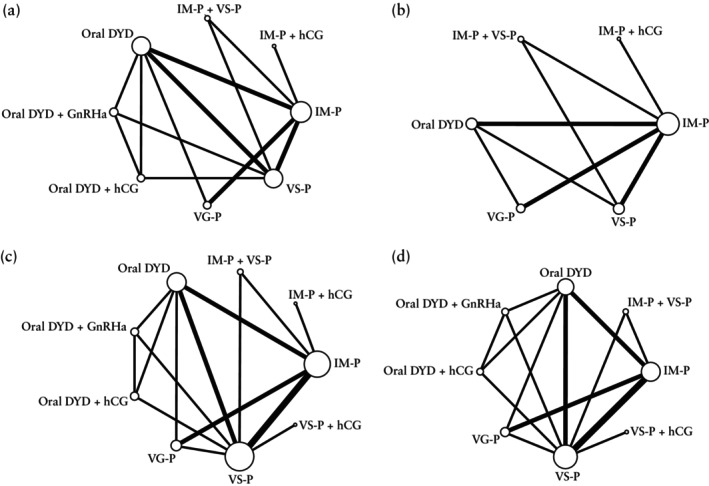
Network maps showing pairwise comparisons of luteal‐phase support protocols analyzed directly in randomized controlled trials for: (a) combined rate of ongoing pregnancy and live birth; (b) live birth rate; (c) clinical pregnancy rate; and (d) pregnancy loss rate. Size of each node is proportional to total number of participants randomized to that treatment. Thickness of connecting lines reflects number of direct comparisons between corresponding treatments. DYD, dydrogesterone; GnRHa, gonadotropin‐releasing hormone agonist; hCG, human chorionic gonadotropin; IM, intramuscular; P, progesterone; VG, vaginal gel; VS, vaginal suppository.

**Figure 3:**
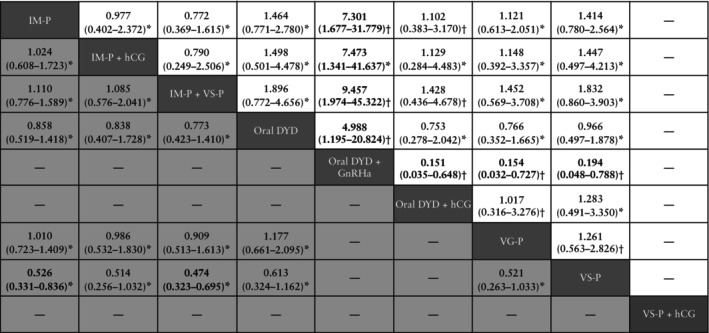
League table for combined rate of live birth and ongoing pregnancy (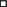) and live birth rate (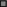), expressed as odds ratios with 95% CI. Data above 1 favor the column‐defining treatment. Data below 1 favor the row‐defining treatment. * and † indicate low and very low certainty of evidence, respectively, according to Confidence in Network Meta‐Analysis (CINeMA) criteria. Bold text indicates *P* < 0.05. DYD, dydrogesterone; GnRHa, gonadotropin‐releasing hormone agonist; hCG, human chorionic gonadotropin; IM, intramuscular; P, progesterone; VG, vaginal gel; VS, vaginal suppository.

The surrogate outcome OPR/LBR was retrieved from six RCTs^22^, ^33^, ^36^, ^37^, ^44^, ^47^ comparing the following LPS protocols: IM progesterone, IM progesterone + hCG, IM progesterone + vaginal suppository progesterone, oral DYD, oral DYD + GnRHa, oral DYD + hCG, vaginal gel progesterone and vaginal suppository progesterone. Five studies^22^, ^33^, ^36^, ^37^, ^44^ reported the LBR, while the OPR was reported by Zarei *et al*.^47^. The network map for OPR/LBR is shown (Figure 2a). The global analysis for inconsistency revealed no evidence of inconsistency (*P* = 0.999), so SIDE‐splitting analysis was not performed. As shown in the league table (Figure 3), the efficacy of oral DYD + GnRHa was significantly higher compared with all other LPS protocols (very low to low certainty of evidence). None of the other comparisons were statistically significant (very low to low certainty of evidence). Certainty of evidence evaluation using CINeMA criteria is provided (Table 1). Based on SUCRA scores, the combination of oral DYD + GnRHa (SUCRA = 97.3%) had the highest probability of being ranked treatment of choice (Table 2).

**Table 1:** Certainty of evidence for combined rate of ongoing pregnancy and live birth using Confidence in Network Meta‐Analysis (CINeMA) criteria

Comparison	Studies (*n*)*	Within‐study bias	Reporting bias	Indirectness	Imprecision	Heterogeneity	Incoherence	Confidence rating
IM‐P *vs* IM‐P + hCG	1	No concerns	Low risk	No concerns	Major concerns	No concerns	Some concerns	Low
IM‐P *vs* IM‐P + VS‐P	1	No concerns	Low risk	No concerns	Major concerns	No concerns	No concerns	Low
IM‐P *vs* oral DYD	2	Some concerns	Low risk	No concerns	Major concerns	No concerns	No concerns	Low
IM‐P *vs* VG‐P	2	Some concerns	Low risk	No concerns	Major concerns	No concerns	No concerns	Low
IM‐P *vs* VS‐P	2	No concerns	Low risk	No concerns	Major concerns	No concerns	No concerns	Low
IM‐P + VS‐P *vs* VS‐P	1	No concerns	Low risk	No concerns	Major concerns	No concerns	No concerns	Low
Oral DYD *vs* oral DYD + GnRHa	1	Some concerns	Low risk	No concerns	No concerns	Major concerns	Major concerns	Very low
Oral DYD *vs* oral DYD + hCG	1	Some concerns	Low risk	No concerns	Major concerns	No concerns	No concerns	Low
Oral DYD *vs* VG‐P	1	Some concerns	Low risk	No concerns	Major concerns	No concerns	No concerns	Low
Oral DYD *vs* VS‐P	2	Some concerns	Low risk	No concerns	Major concerns	No concerns	No concerns	Low
Oral DYD + GnRHa *vs* oral DYD + hCG	1	Some concerns	Low risk	No concerns	No concerns	Major concerns	Some concerns	Very low
Oral DYD + GnRHa *vs* VS‐P	1	Some concerns	Low risk	No concerns	No concerns	Major concerns	Major concerns	Very low
Oral DYD + hCG *vs* VS‐P	1	Some concerns	Low risk	No concerns	Major concerns	No concerns	No concerns	Low
IM‐P *vs* oral DYD + GnRHa	0	Some concerns	Low risk	No concerns	No concerns	Major concerns	Some concerns	Very low
IM‐P *vs* oral DYD + hCG	0	Some concerns	Low risk	No concerns	Major concerns	No concerns	Some concerns	Very low
IM‐P + hCG *vs* IM‐P + VS‐P	0	No concerns	Low risk	No concerns	Major concerns	No concerns	Some concerns	Low
IM‐P + hCG *vs* oral DYD	0	No concerns	Low risk	No concerns	Major concerns	No concerns	Some concerns	Low
IM‐P + hCG *vs* oral DYD + GnRHa	0	No concerns	Low risk	No concerns	No concerns	Major concerns	Some concerns	Low
IM‐P + hCG *vs* oral DYD + hCG	0	No concerns	Low risk	No concerns	Major concerns	No concerns	Some concerns	Low
IM‐P + hCG *vs* VG‐P	0	No concerns	Low risk	No concerns	Major concerns	No concerns	Some concerns	Low
IM‐P + hCG *vs* VS‐P	0	No concerns	Low risk	No concerns	Major concerns	No concerns	Some concerns	Low
IM‐P + VS‐P *vs* oral DYD	0	No concerns	Low risk	No concerns	Major concerns	No concerns	Some concerns	Low
IM‐P + VS‐P *vs* oral DYD + GnRHa	0	Some concerns	Low risk	No concerns	No concerns	Major concerns	Some concerns	Very low
IM‐P + VS‐P *vs* oral DYD + hCG	0	Some concerns	Low risk	No concerns	Major concerns	No concerns	Some concerns	Very low
IM‐P + VS‐P *vs* VG‐P	0	No concerns	Low risk	No concerns	Major concerns	No concerns	Some concerns	Low
Oral DYD + GnRHa *vs* VG‐P	0	Some concerns	Low risk	No concerns	No concerns	Major concerns	Some concerns	Very low
Oral DYD + hCG *vs* VG‐P	0	Some concerns	Low risk	No concerns	Major concerns	No concerns	Some concerns	Very low
VG‐P *vs* VS‐P	0	Some concerns	Low risk	No concerns	Major concerns	No concerns	Some concerns	Very low

* Studies that performed direct pairwise comparison.

DYD, dydrogesterone; GnRHa, gonadotropin‐releasing hormone agonist; hCG, human chorionic gonadotropin; IM, intramuscular; P, progesterone; VG, vaginal gel; VS, vaginal suppository.

**Table 2:** Surface under the cumulative ranking curve area (SUCRA) table showing likelihood of various luteal‐phase support protocols being ranked treatment of choice for study outcomes

	OPR/LBR		LBR		CPR		PLR	
Rank	Protocol	SUCRA (%)	Protocol	SUCRA (%)	Protocol	SUCRA (%)	Protocol	SUCRA (%)
1	Oral DYD + GnRHa	97.3	VS‐P	89.7	VS‐P + hCG	33.7	IM‐P + VS‐P	51.4
2	IM‐P + hCG	0.8	Oral DYD	5.7	IM‐P + hCG	18.4	VS‐P + hCG	17.7
3	VS‐P	0.6	IM‐P + hCG	2.6	Oral DYD	16.1	Oral DYD + hCG	12.0
4	Oral DYD	0.6	VG‐P	2.0	Oral DYD + hCG	15.7	Oral DYD + GnRHa	9.7
5	Oral DYD + hCG	0.4	IM‐P	0	VS‐P	8.9	VG‐P	6.8
6	VG‐P	0.3	IM‐P + VS‐P	0	VG‐P	5.6	IM‐P	1.7
7	IM‐P	0	—	—	Oral P + GnRHa	0.8	Oral DYD	0.7
8	IM‐P + VS‐P	0	—	—	IM‐P + VS‐P	0.7	VS‐P	0
9	—	—	—	—	IM‐P	0.1	—	—

CPR, clinical pregnancy rate; DYD, dydrogesterone; GnRHa, gonadotropin‐releasing hormone agonist; hCG, human chorionic gonadotropin; IM, intramuscular; LBR, live birth rate; OPR/LBR, combined rate of ongoing pregnancy and live birth; P, progesterone; PLR, pregnancy loss rate; VG, vaginal gel; VS, vaginal suppository.

We assessed the LBR alone (without supplementing it with the OPR), restricting the analysis to five studies^22^, ^33^, ^36^, ^37^, ^44^ which compared the following LPS protocols: IM progesterone, IM progesterone + hCG, IM progesterone + vaginal suppository progesterone, oral DYD, vaginal gel progesterone and vaginal suppository progesterone. The network map for LBR is provided (Figure 2b). No evidence of inconsistency was noted on global analysis (*P* = 0.999), so SIDE‐splitting analysis was not performed. IM progesterone (OR, 0.53 (95% CI, 0.33–0.84); low certainty of evidence) and IM progesterone + vaginal suppository progesterone (OR, 0.47 (95% CI, 0.32–0.69); low certainty of evidence) were significantly less efficacious compared with vaginal suppository progesterone alone (Figure 3). No other significant differences were noted in the league table (low certainty of evidence). Certainty of evidence evaluation using CINeMA criteria is shown in Appendix S3. SUCRA analysis showed that vaginal suppository progesterone (SUCRA = 89.7%) had the highest likelihood of being the top‐ranked approach (Table 2).

#### Clinical pregnancy rate

**Figure 4:**
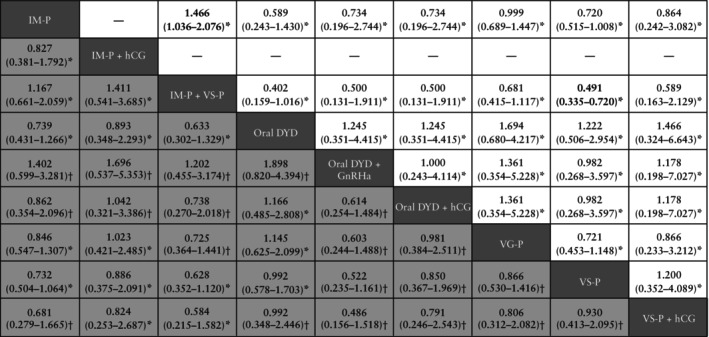
League table for pregnancy loss rate (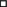) and clinical pregnancy rate (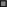), expressed as odds ratios with 95% CI. Data above 1 favor the column‐defining treatment. Data below 1 favor the row‐defining treatment. * and † indicate low and very low certainty of evidence, respectively, according to CINeMA criteria. Bold text indicates *P* < 0.05. DYD, dydrogesterone; GnRHa, gonadotropin‐releasing hormone agonist; hCG, human chorionic gonadotropin; IM, intramuscular; P, progesterone; VG, vaginal gel; VS, vaginal suppository.

All 10 RCTs^22^, ^33^, ^34^, ^35^, ^36^, ^37^, ^40^, ^41^, ^44^, ^47^ estimated the CPR. The following LPS protocols were evaluated: IM progesterone, IM progesterone + hCG, IM progesterone + vaginal suppository progesterone, oral DYD, oral DYD + GnRHa, oral DYD + hCG, vaginal gel progesterone, vaginal suppository progesterone and vaginal suppository progesterone + hCG. The network of direct comparisons is provided (Figure 2c). Inconsistency was not found in the global analysis (*P* = 0.057). Nevertheless, closed‐loop SIDE‐splitting analysis was performed because of the complexity of the network geometry, and some significant inconsistency was noted (*P* < 0.05) (Table S3). The league table shows that there were no significant differences between protocols on direct and indirect pairwise comparisons (very low to low certainty of evidence) (Figure 4). Certainty of evidence evaluation using CINeMA criteria is shown in Appendix S3. According to SUCRA ranking, vaginal suppository progesterone + hCG (SUCRA = 33.7%), IM progesterone + hCG (SUCRA = 18.4%), oral DYD (SUCRA = 16.1%) and oral DYD + hCG (SUCRA = 15.7%) had the highest chances of being ranked treatment of choice among the LPS protocols evaluated by the RCTs (Table 2).

#### Pregnancy loss rate

PLR was retrieved from eight RCTs^33^, ^35^, ^36^, ^37^, ^40^, ^41^, ^44^, ^47^. The following LPS protocols were evaluated: IM progesterone, IM progesterone + vaginal suppository progesterone, oral DYD, oral DYD + GnRHa, oral DYD + hCG, vaginal gel progesterone, vaginal suppository progesterone and vaginal suppository progesterone + hCG. The network of direct comparisons is provided (Figure 2d). No evidence of inconsistency was noted on global analysis (*P* = 0.999). Nevertheless, closed‐loop SIDE‐splitting analysis was performed because of the complexity of the network geometry, and no inconsistency was noted (Table S3). The league table of direct and indirect comparisons shows that IM progesterone + vaginal suppository progesterone was significantly more efficacious compared with IM progesterone alone (OR, 1.47 (95% CI, 1.04–2.08); low certainty of evidence), whereas vaginal suppository progesterone alone was significantly less efficacious compared with IM progesterone + vaginal suppository progesterone (OR, 0.49 (95% CI, 0.34–0.72); low certainty of evidence) (Figure 4). No other significant differences were reported (low certainty of evidence). Certainty of evidence evaluation using CINeMA criteria is shown in Appendix S3. Based on SUCRA analysis, IM progesterone + vaginal suppository progesterone had the highest chance of being the top‐ranked treatment for minimizing pregnancy loss (SUCRA = 51.4%), followed by vaginal suppository progesterone + hCG (SUCRA = 17.7%) (Table 2).

#### Sensitivity analysis

Significant (*P* < 0.05) inconsistencies were noted in the closed‐loop SIDE‐splitting analysis for CPR, so in order to reduce the inconsistency and explain the differences among the RCTs for this outcome, the potential sources of heterogeneity between the studies were assessed using predefined sensitivity analyses (Appendix S4). No residual inconsistency was detected after the sensitivity analyses.

## DISCUSSION

### Summary of evidence

There is very‐low‐to‐low‐certainty evidence that the combination of oral DYD + GnRHa was the LPS protocol with the greatest probability of achieving the highest OPR/LBR. However, when restricting the outcome to LBR alone, vaginal suppository progesterone demonstrated the highest likelihood of being the top‐ranked LPS protocol (low certainty of evidence). This discrepancy indicates some inconsistency in the rankings depending on the outcome definition, which should be considered when interpreting the findings. The CPR did not differ significantly between the LPS protocols evaluated (very low to low certainty of evidence). However, IM progesterone + vaginal suppository progesterone appeared to perform better in minimizing pregnancy loss (low certainty of evidence).

### Comparison with existing literature

Several LPS protocols have been reported to increase the CPR and OPR/LBR, and to reduce the PLR; nevertheless, the evidence is inconsistent. A 2010 Cochrane review concluded that the available evidence was insufficient to recommend one particular LPS protocol over another^48^. Previous work found that vaginal gel progesterone yielded similar reproductive outcomes compared with oral DYD^46^ and IM progesterone^44^. Similarly, treatment with oral DYD, vaginal gel progesterone or IM progesterone during HRT–FET cycles resulted in similar CPR and LBR in a three‐arm RCT^36^. Moreover, comparable LBR and PLR were obtained using SC progesterone and vaginal suppository progesterone supplementation^43^. However, when compared with IM progesterone, vaginal suppository progesterone alone appears to be less effective in sustaining embryo implantation and early survival^24^.

In our analysis, combined regimens that included GnRHa or hCG achieved the highest ranks in the SUCRA analyses for OPR/LBR and CPR. Supplementation with these agents remains less investigated, more controversial and, at the same time, more intriguing. The use of GnRHa for LPS has already been tested in fresh embryo transfer cycles, yielding contradictory results in terms of implantation rate and LBR^49^. However, a Cochrane systematic review reported higher OPR or LBR after GnRHa administration in the luteal phase of fresh embryo transfer cycles^50^. Yet, the evidence in HRT–FET cycles is currently limited. GnRHa could exert a beneficial effect in different ways. It could potentially bolster the corpus luteum by triggering the release of luteinizing hormone from the pituitary gland^51^. Moreover, gonadotropin‐releasing hormone and its receptors are found in the endometrium during the implantation window, as well as on preimplantation embryos of both rodents and humans^52^, ^53^. Regarding the addition of GnRHa to progesterone supplementation in LPS for HRT–FET, a recent retrospective analysis found improved outcomes in patients treated with GnRHa compared with those who received conventional hormone therapy^23^, while a prospective interventional pilot study found a statistically non‐significant increase in LBR and a decrease in PLR when GnRHa was compared with standard HRT^38^. Based on the limited evidence from the present network meta‐analysis, the addition of GnRHa to oral DYD, or the use of vaginal suppository progesterone alone, may improve the outcome of HRT–FET cycles.

Several studies have investigated the function of hCG in HRT–FET cycles and have yielded contradictory results, probably due to limited power with small sample sizes^41^, ^47^. SUCRA analysis in the present study shows that oral DYD + hCG could be a treatment of choice for achieving a higher CPR; nevertheless, two RCTs did not find any benefit from the addition of hCG compared with controls treated with a conventional HRT protocol^22^, ^41^. According to our analysis, vaginal suppository progesterone + hCG and IM progesterone + hCG could be valid alternatives.

### Strengths and limitations

This network meta‐analysis has several limitations that should be acknowledged. According to the CINeMA framework, many comparisons were rated as having low or very low certainty of evidence. Some trials lacked adequate randomization and allocation concealment, and most had inadequate blinding of participants, personnel and/or outcome assessment, which could impact the robustness of the findings. The poor agreement regarding the superiority of LPS protocols might be a matter of imprecision, reducing the certainty of the evaluated evidence. Network inconsistencies within closed loops further reduced the certainty for specific comparisons. Additionally, heterogeneity across studies remained a concern because of variations in inclusion/exclusion criteria and the absence of sufficient data on critical covariates (e.g. progesterone level on the day of the trigger, estrogen level at the time of trigger, maternal body mass index and the use of preimplantation genetic testing). Sensitivity analyses excluding studies with a mean maternal age over 35 years, those that transferred cleavage‐stage embryos, and those that did not report endometrial thickness at the time of embryo transfer confirmed the main findings and did not show any residual inconsistency, but other unmeasured confounders may still contribute to heterogeneity. These constraints highlight the inherent limitations of the current literature, as certain subgroups and variables were not represented or measured in specific treatment conditions.

Nonetheless, this network meta‐analysis has several strengths. The lack of evidence for publication bias suggests that the results are less likely to be influenced by selective reporting. Additionally, the study covers a broad range of geographic regions, increasing its generalizability, while the large number of enrolled women enhances the robustness of the findings. Lastly, our use of the CINeMA approach to evaluate certainty of evidence ensures methodological rigor and transparency in the grading of findings.

### Clinical implications and future directions

The heterogeneity of our findings suggests that patient‐specific factors, such as previous pregnancy loss, progesterone level and tolerance to specific administration routes, should be considered when selecting a LPS protocol. The combination of IM progesterone with vaginal suppository progesterone appears beneficial in reducing the PLR, indicating its potential use for patients with a history of implantation failure or repeat pregnancy loss. However, the pain associated with the administration of IM progesterone and the risk of injection‐site complications should be weighed against the benefits. Oral DYD, which showed promising efficacy for the OPR/LBR when combined with GnRHa, may offer a more patient‐friendly alternative to IM progesterone, improving both adherence and patient experience^54^. The absence of statistically significant differences among LPS protocols for CPR suggests that clinical pregnancy may not be the primary factor influenced by LPS regimen selection. Instead, the future focus should be on optimizing LPS to sustain pregnancy postimplantation. The role of GnRHa and hCG in improving pregnancy outcome also requires further exploration, as the current evidence is limited and inconsistent. Similarly, future studies should investigate individualized LPS strategies based on progesterone level, uterine issues and genetic factors influencing progesterone metabolism. A precision medicine approach could help to tailor LPS protocols to specific patient subgroups for enhanced efficacy. In addition, economic analyses comparing the cost‐effectiveness of different LPS regimens should be conducted to identify financially sustainable and widely accessible treatment options.

### Conclusion

This network meta‐analysis provides very‐low‐to‐low‐certainty evidence that the combination of oral DYD and GnRHa, as well as vaginal suppository progesterone alone, may be among the most effective LPS protocols for improving live‐birth outcomes in HRT–FET cycles. For the mitigation of pregnancy loss, IM progesterone + vaginal suppository progesterone seems to be more effective compared with other LPS protocols. However, inconsistencies across different outcome measures, very low to low certainty of evidence and potential variation in patient response underscore the need for further robust research. Additional high‐quality RCTs are necessary to define the optimal LPS strategy, develop tailored treatment approaches, and evaluate long‐term maternal and neonatal outcomes of assisted reproductive technology practices.

## Supporting information


**Appendix S1** Search strategy for each database

**Appendix S2** Studies excluded after full‐text assessment and reason for exclusion

**Appendix S3** Certainty of evidence evaluation for live birth rate, clinical pregnancy rate and pregnancy loss rate using CINeMA criteria

**Appendix S4** Sensitivity analyses for study outcomes

**Figure S1** Risk‐of‐bias assessment for included studies. Results are shown for each individual study (a) and according to each risk‐of‐bias item (b). +, low risk of bias; −, high risk of bias; ?, unclear risk of bias.

**Figure S2** Funnel plot for clinical pregnancy rate.

**Table S1** Description of Cochrane risk‐of‐bias tool version 1 (RoB 1) and results of quality assessment of included studies using RoB 1

**Table S2** Characteristics of studies included in quantitative analysis

**Table S3** Evaluation of local inconsistency for study outcomes using separating indirect from direct evidence (SIDE)‐splitting analysis

## Data Availability

The data that support the findings of this study are available from the corresponding author upon reasonable request.
