# Supplementary material for: Reproductive outcome after frozen embryo transfer with hormone replacement therapy according to luteal‐phase support protocol: systematic review and network meta‐analysis of randomized controlled trials
Source: Ultrasound Obstet Gynecol. 2025 Aug 1;66(4):422–32. doi: 10.1002/uog.29302 (PMC12488206; doi:10.1002/uog.29302)
Supplement: Supplementary file 1 — Appendix S1 Search strategy for each database [file UOG-66-422-s006.docx]

**Appendix S1** Search strategy

**EMBASE**

('luteal phase support'/exp OR 'luteal phase support' OR (luteal AND phase AND ('support'/exp OR support))) AND ('hormone replacement therapy'/exp OR 'hormone replacement therapy' OR (('hormone'/exp OR hormone) AND ('replacement'/exp OR replacement) AND ('therapy'/exp OR therapy)) OR 'artificial cycle' OR (artificial AND cycle)) AND ('frozen-thawed embryo-transfer' OR ('frozen thawed' AND ('embryo transfer'/exp OR 'embryo transfer')) OR 'frozen embryo transfer'/exp OR 'frozen embryo transfer' OR (frozen AND ('embryo'/exp OR embryo) AND ('transfer'/exp OR transfer)))

**MEDLINE (accessed through Pubmed)**

("luteal phase"[MeSH Terms] OR ("luteal"[All Fields] AND "phase"[All Fields]) OR "luteal phase"[All Fields]) AND ("support"[All Fields] OR "support s"[All Fields] OR "supported"[All Fields] OR "supporter"[All Fields] OR "supporter s"[All Fields] OR "supporters"[All Fields] OR "supporting"[All Fields] OR "supportive"[All Fields] OR "supportiveness"[All Fields] OR "supports"[All Fields]) AND ("hormone replacement therapy"[MeSH Terms] OR ("hormone"[All Fields] AND "replacement"[All Fields] AND "therapy"[All Fields]) OR "hormone replacement therapy"[All Fields] OR (("artificial"[All Fields] OR "artificially"[All Fields]) AND ("bicycling"[MeSH Terms] OR "bicycling"[All Fields] OR "cycling"[All Fields] OR "cycle"[All Fields] OR "cycle s"[All Fields] OR "cycled"[All Fields] OR "cycles"[All Fields] OR "cyclings"[All Fields]))) AND (("frozen-thawed"[All Fields] AND ("embryo transfer"[MeSH Terms] OR ("embryo"[All Fields] AND "transfer"[All Fields]) OR "embryo transfer"[All Fields])) OR (("freezing"[MeSH Terms] OR "freezing"[All Fields] OR "frozen"[All Fields]) AND ("embryo transfer"[MeSH Terms] OR ("embryo"[All Fields] AND "transfer"[All Fields]) OR "embryo transfer"[All Fields])))

**SCOPUS**

TITLE-ABS-KEY ( luteal AND phase AND support AND ( hormone AND replacement AND therapy OR artificial AND cycle ) AND ( frozen-thawed AND embryo-transfer OR frozen AND embryo AND transfer ) )

**COCHRANE at CENTRAL**

luteal phase support and (hormone replacement therapy or artificial cycle) and (frozen-thawed embryo-transfer or frozen embryo transfer):ti,ab,kw

**CINAHL / PsycINFO / AMED / PsycExtra /NTIS (accessed through EBSCO – IDEM for Italian Universities)**

luteal phase support and (hormone replacement therapy or artificial cycle) and (frozen-thawed embryo-transfer or frozen embryo transfer) AND Cerca anche nel testo completo degli articoli; Applica argomenti equivalenti

**LILACS**

luteal phase support and (hormone replacement therapy or artificial cycle) and (frozen-thawed embryo-transfer or frozen embryo transfer)

**Scielo.br**

luteal phase support and (hormone replacement therapy or artificial cycle) and (frozen-thawed embryo-transfer or frozen embryo transfer)

**Clinicaltrials.gov / ICTRP (accessed through CENTRAL)**

luteal phase support and (hormone replacement therapy or artificial cycle) and (frozen-thawed embryo-transfer or frozen embryo transfer) AND (endometriosis):ti,ab,kw

**PROSPERO**

luteal phase support and (hormone replacement therapy or artificial cycle) and (frozen-thawed embryo-transfer or frozen embryo transfer)
