# Supplementary material for: Reproductive outcome after frozen embryo transfer with hormone replacement therapy according to luteal‐phase support protocol: systematic review and network meta‐analysis of randomized controlled trials
Source: Ultrasound Obstet Gynecol. 2025 Aug 1;66(4):422–32. doi: 10.1002/uog.29302 (PMC12488206; doi:10.1002/uog.29302)
Supplement: Supplementary file 2 — Appendix S2 Studies excluded after full‐text assessment and reason for exclusion [file UOG-66-422-s005.docx]

**Appendix S2** Studies excluded after full-text assessment and reason for exclusion

| **Article** | **Reason for exclusion** |
| --- | --- |
| Vidal A, Dhakal C, Werth N, Weiss JM, Lehnick D and Kohl Schwartz AS (2023) Supplementary dydrogesterone is beneficial as luteal phase support in artificial frozen-thawed embryo transfer cycles compared to micronized progesterone alone.  Front. Endocrinol. 14:1128564. doi: 10.3389/fendo.2023.1128564 | Post hoc grouping; overlapping treatment in at least two cohorts |
|  |  |
| Chang WS, Lin PH, Li CJ, Chern CU, Chen YC, Lin LT, Tsui KH. Additional single dose GnRH agonist during luteal phase support may improve live birth rate in GnRHa-HRT frozen-thawed embryo transfer cycle: a retrospective cohort study. BMC Pregnancy Childbirth. 2023 Mar 14;23(1):174. doi: 10.1186/s12884-023-05491-y. | Same route of administration with add-on strategy |
