# Supplementary material for: Reproductive outcome after frozen embryo transfer with hormone replacement therapy according to luteal‐phase support protocol: systematic review and network meta‐analysis of randomized controlled trials
Source: Ultrasound Obstet Gynecol. 2025 Aug 1;66(4):422–32. doi: 10.1002/uog.29302 (PMC12488206; doi:10.1002/uog.29302)
Supplement: Supplementary file 3 — Appendix S3 Certainty of evidence evaluation for live birth rate, clinical pregnancy rate and pregnancy loss rate using CINeMA criteria [file UOG-66-422-s001.docx]

**Appendix S3**. Certainty of evidence evaluation for live birth rate, clinical pregnancy rate and pregnancy loss rate using CINeMA criteria

Live birth rate

| **Comparison** | **Number of studies** | **Within-study bias** | **Reporting bias** | **Indirectness** | **Imprecision** | **Heterogeneity** | **Incoherence** | **Confidence rating** |
| --- | --- | --- | --- | --- | --- | --- | --- | --- |
| **IM P:IM P +  HCG** | 1 | No concerns | Low risk | No concerns | Major concerns | No concerns | No concerns | Low |
| **IM P:IM P + Vaginal suppositories P** | 1 | No concerns | Low risk | No concerns | Major concerns | No concerns | No concerns | Low |
| **IM P:Oral DYD** | 2 | No concerns | Low risk | No concerns | Major concerns | No concerns | No concerns | Low |
| **IM P:Vaginal gel P** | 2 | Some concerns | Low risk | No concerns | Major concerns | No concerns | No concerns | Low |
| **IM P:Vaginal suppositories P** | 2 | No concerns | Low risk | No concerns | No concerns | Major concerns | No concerns | Low |
| **IM P + Vaginal suppositories P:Vaginal suppositories P** | 1 | No concerns | Low risk | No concerns | No concerns | Major concerns | No concerns | Low |
| **Oral DYD:Vaginal gel P** | 1 | Some concerns | Low risk | No concerns | Major concerns | No concerns | No concerns | Low |
| **Oral DYD:Vaginal suppositories P** | 1 | No concerns | Low risk | No concerns | Major concerns | No concerns | No concerns | Low |
| **IM P +  HCG:IM P + Vaginal suppositories P** | 0 | No concerns | Low risk | No concerns | Major concerns | No concerns | No concerns | Low |
| **IM P +  HCG:Oral DYD** | 0 | No concerns | Low risk | No concerns | Major concerns | No concerns | No concerns | Low |
| **IM P +  HCG:Vaginal gel P** | 0 | No concerns | Low risk | No concerns | Major concerns | No concerns | No concerns | Low |
| **IM P +  HCG:Vaginal suppositories P** | 0 | No concerns | Low risk | No concerns | No concerns | Major concerns | No concerns | Low |
| **IM P + Vaginal suppositories P:Oral DYD** | 0 | No concerns | Low risk | No concerns | Major concerns | No concerns | No concerns | Low |
| **IM P + Vaginal suppositories P:Vaginal gel P** | 0 | No concerns | Low risk | No concerns | Major concerns | No concerns | No concerns | Low |
| **Vaginal gel P:Vaginal suppositories P** | 0 | No concerns | Low risk | No concerns | No concerns | Major concerns | No concerns | Low |

Clinical pregnancy rate

| **Comparison** | **Number of studies** | **Within-study bias** | **Reporting bias** | **Indirectness** | **Imprecision** | **Heterogeneity** | **Incoherence** | **Confidence rating** |
| --- | --- | --- | --- | --- | --- | --- | --- | --- |
| **IM P:IM P +  HCG** | 1 | No concerns | Low risk | No concerns | Major concerns | No concerns | Some concerns | Low |
| **IM P:IM P + Vaginal suppositories P** | 1 | No concerns | Low risk | No concerns | Major concerns | No concerns | No concerns | Low |
| **IM P:Oral DYD** | 2 | Some concerns | Low risk | No concerns | Major concerns | No concerns | No concerns | Low |
| **IM P:Vaginal gel P** | 2 | Some concerns | Low risk | No concerns | Major concerns | No concerns | No concerns | Low |
| **IM P:Vaginal suppositories P** | 4 | No concerns | Low risk | No concerns | Major concerns | No concerns | No concerns | Low |
| **IM P + Vaginal suppositories P:Vaginal suppositories P** | 1 | No concerns | Low risk | No concerns | Major concerns | No concerns | No concerns | Low |
| **Oral DYD:Oral DYD +  GnRHa** | 1 | Some concerns | Low risk | No concerns | Major concerns | No concerns | Major concerns | Very Low |
| **Oral DYD:Oral DYD +  HCG** | 1 | Some concerns | Low risk | No concerns | Major concerns | No concerns | No concerns | Low |
| **Oral DYD:Vaginal gel P** | 1 | Some concerns | Low risk | No concerns | Major concerns | No concerns | No concerns | Low |
| **Oral DYD:Vaginal suppositories P** | 2 | Some concerns | Low risk | No concerns | Major concerns | No concerns | No concerns | Low |
| **Oral DYD +  GnRHa:Oral DYD +  HCG** | 1 | Some concerns | Low risk | No concerns | Major concerns | No concerns | Some concerns | Very Low |
| **Oral DYD +  GnRHa:Vaginal suppositories P** | 1 | Some concerns | Low risk | No concerns | Major concerns | No concerns | Major concerns | Very Low |
| **Oral DYD +  HCG:Vaginal suppositories P** | 1 | Some concerns | Low risk | No concerns | Major concerns | No concerns | Major concerns | Very Low |
| **Vaginal gel P:Vaginal suppositories P** | 1 | Some concerns | Low risk | No concerns | Major concerns | No concerns | No concerns | Very Low |
| **Vaginal suppositories P:Vaginal suppositories P  +  HCG** | 1 | Major concerns | Low risk | No concerns | Major concerns | No concerns | Some concerns | Very Low |
| **IM P:Oral DYD +  GnRHa** | 0 | Some concerns | Low risk | No concerns | Major concerns | No concerns | Some concerns | Very Low |
| **IM P:Oral DYD +  HCG** | 0 | Some concerns | Low risk | No concerns | Major concerns | No concerns | Some concerns | Very Low |
| **IM P:Vaginal suppositories P  +  HCG** | 0 | Major concerns | Low risk | No concerns | Major concerns | No concerns | Some concerns | Very Low |
| **IM P +  HCG:IM P + Vaginal suppositories P** | 0 | No concerns | Low risk | No concerns | Major concerns | No concerns | Some concerns | Low |
| **IM P +  HCG:Oral DYD** | 0 | No concerns | Low risk | No concerns | Major concerns | No concerns | Some concerns | Low |
| **IM P +  HCG:Oral DYD +  GnRHa** | 0 | Some concerns | Low risk | No concerns | Major concerns | No concerns | Some concerns | Very Low |
| **IM P +  HCG:Oral DYD +  HCG** | 0 | Some concerns | Low risk | No concerns | Major concerns | No concerns | Some concerns | Very Low |
| **IM P +  HCG:Vaginal gel P** | 0 | No concerns | Low risk | No concerns | Major concerns | No concerns | Some concerns | Low |
| **IM P +  HCG:Vaginal suppositories P** | 0 | No concerns | Low risk | No concerns | Major concerns | No concerns | Some concerns | Low |
| **IM P +  HCG:Vaginal suppositories P  +  HCG** | 0 | No concerns | Low risk | No concerns | Major concerns | No concerns | Some concerns | Low |
| **IM P + Vaginal suppositories P:Oral DYD** | 0 | No concerns | Low risk | No concerns | Major concerns | No concerns | Some concerns | Low |
| **IM P + Vaginal suppositories P:Oral DYD +  GnRHa** | 0 | Some concerns | Low risk | No concerns | Major concerns | No concerns | Some concerns | Very Low |
| **IM P + Vaginal suppositories P:Oral DYD +  HCG** | 0 | Some concerns | Low risk | No concerns | Major concerns | No concerns | Some concerns | Very Low |
| **IM P + Vaginal suppositories P:Vaginal gel P** | 0 | Some concerns | Low risk | No concerns | Major concerns | No concerns | Some concerns | Very Low |
| **IM P + Vaginal suppositories P:Vaginal suppositories P  +  HCG** | 0 | No concerns | Low risk | No concerns | Major concerns | No concerns | Some concerns | Low |
| **Oral DYD:Vaginal suppositories P  +  HCG** | 0 | Major concerns | Low risk | No concerns | Major concerns | No concerns | Some concerns | Very Low |
| **Oral DYD +  GnRHa:Vaginal gel P** | 0 | Some concerns | Low risk | No concerns | Major concerns | No concerns | Some concerns | Very Low |
| **Oral DYD +  GnRHa:Vaginal suppositories P  +  HCG** | 0 | Some concerns | Low risk | No concerns | Major concerns | No concerns | Some concerns | Very Low |
| **Oral DYD +  HCG:Vaginal gel P** | 0 | Some concerns | Low risk | No concerns | Major concerns | No concerns | Some concerns | Very Low |
| **Oral DYD +  HCG:Vaginal suppositories P  +  HCG** | 0 | Some concerns | Low risk | No concerns | Major concerns | No concerns | Some concerns | Very Low |
| **Vaginal gel P:Vaginal suppositories P  +  HCG** | 0 | Some concerns | Low risk | No concerns | Major concerns | No concerns | Some concerns | Very Low |

Pregnancy loss rate

| **Comparison** | **Number of studies** | **Within-study bias** | **Reporting bias** | **Indirectness** | **Imprecision** | **Heterogeneity** | **Incoherence** | **Confidence rating** |
| --- | --- | --- | --- | --- | --- | --- | --- | --- |
| **IM P:IM P + Vaginal suppositories P** | 1 | No concerns | Low risk | No concerns | No concerns | Major concerns | No concerns | Low |
| **IM P:Oral DYD** | 2 | Some concerns | Low risk | No concerns | Major concerns | No concerns | No concerns | Low |
| **IM P:Vaginal gel P** | 2 | Some concerns | Low risk | No concerns | Major concerns | No concerns | No concerns | Low |
| **IM P:Vaginal suppositories P** | 3 | No concerns | Low risk | No concerns | Major concerns | No concerns | No concerns | Low |
| **IM P + Vaginal suppositories P:Vaginal suppositories P** | 1 | No concerns | Low risk | No concerns | No concerns | No concerns | No concerns | Low |
| **Oral DYD:Oral DYD +  GnRHa** | 1 | Some concerns | Low risk | No concerns | Major concerns | No concerns | No concerns | Low |
| **Oral DYD:Oral DYD +  HCG** | 1 | Some concerns | Low risk | No concerns | Major concerns | No concerns | No concerns | Low |
| **Oral DYD:Vaginal gel P** | 1 | Some concerns | Low risk | No concerns | Major concerns | No concerns | No concerns | Low |
| **Oral DYD:Vaginal suppositories P** | 2 | Some concerns | Low risk | No concerns | Major concerns | No concerns | No concerns | Low |
| **Oral DYD +  GnRHa:Oral DYD +  HCG** | 1 | Some concerns | Low risk | No concerns | Major concerns | No concerns | No concerns | Low |
| **Oral DYD +  GnRHa:Vaginal suppositories P** | 1 | Some concerns | Low risk | No concerns | Major concerns | No concerns | No concerns | Low |
| **Oral DYD +  HCG:Vaginal suppositories P** | 1 | Some concerns | Low risk | No concerns | Major concerns | No concerns | No concerns | Low |
| **Vaginal gel P:Vaginal suppositories P** | 1 | Some concerns | Low risk | No concerns | Major concerns | No concerns | No concerns | Low |
| **Vaginal suppositories P:Vaginal suppositories P  +  HCG** | 1 | Major concerns | Low risk | No concerns | Major concerns | No concerns | No concerns | Low |
| **IM P:Oral DYD +  GnRHa** | 0 | Some concerns | Low risk | No concerns | Major concerns | No concerns | No concerns | Low |
| **IM P:Oral DYD +  HCG** | 0 | Some concerns | Low risk | No concerns | Major concerns | No concerns | No concerns | Low |
| **IM P:Vaginal suppositories P  +  HCG** | 0 | No concerns | Low risk | No concerns | Major concerns | No concerns | No concerns | Low |
| **IM P + Vaginal suppositories P:Oral DYD** | 0 | No concerns | Low risk | No concerns | Major concerns | No concerns | No concerns | Low |
| **IM P + Vaginal suppositories P:Oral DYD +  GnRHa** | 0 | Some concerns | Low risk | No concerns | Major concerns | No concerns | No concerns | Low |
| **IM P + Vaginal suppositories P:Oral DYD +  HCG** | 0 | Some concerns | Low risk | No concerns | Major concerns | No concerns | No concerns | Low |
| **IM P + Vaginal suppositories P:Vaginal gel P** | 0 | No concerns | Low risk | No concerns | Major concerns | No concerns | No concerns | Low |
| **IM P + Vaginal suppositories P:Vaginal suppositories P  +  HCG** | 0 | No concerns | Low risk | No concerns | Major concerns | No concerns | No concerns | Low |
| **Oral DYD:Vaginal suppositories P  +  HCG** | 0 | Some concerns | Low risk | No concerns | Major concerns | No concerns | No concerns | Low |
| **Oral DYD +  GnRHa:Vaginal gel P** | 0 | Some concerns | Low risk | No concerns | Major concerns | No concerns | No concerns | Low |
| **Oral DYD +  GnRHa:Vaginal suppositories P  +  HCG** | 0 | Some concerns | Low risk | No concerns | Major concerns | No concerns | No concerns | Low |
| **Oral DYD +  HCG:Vaginal gel P** | 0 | Some concerns | Low risk | No concerns | Major concerns | No concerns | No concerns | Low |
| **Oral DYD +  HCG:Vaginal suppositories P  +  HCG** | 0 | Some concerns | Low risk | No concerns | Major concerns | No concerns | No concerns | Low |
| **Vaginal gel P:Vaginal suppositories P  +  HCG** | 0 | Some concerns | Low risk | No concerns | Major concerns | No concerns | No concerns | Low |
