# Supplementary material for: Reproductive outcome after frozen embryo transfer with hormone replacement therapy according to luteal‐phase support protocol: systematic review and network meta‐analysis of randomized controlled trials
Source: Ultrasound Obstet Gynecol. 2025 Aug 1;66(4):422–32. doi: 10.1002/uog.29302 (PMC12488206; doi:10.1002/uog.29302)
Supplement: Supplementary file 4 — Appendix S4 Sensitivity analyses for study outcomes [file UOG-66-422-s009.docx]

**Appendix S4.** Sensitivity analyses for study outcomes

**1) Sensitivity analysis excluding studies with mean age over 35**

- - CPR

*Network map*

**
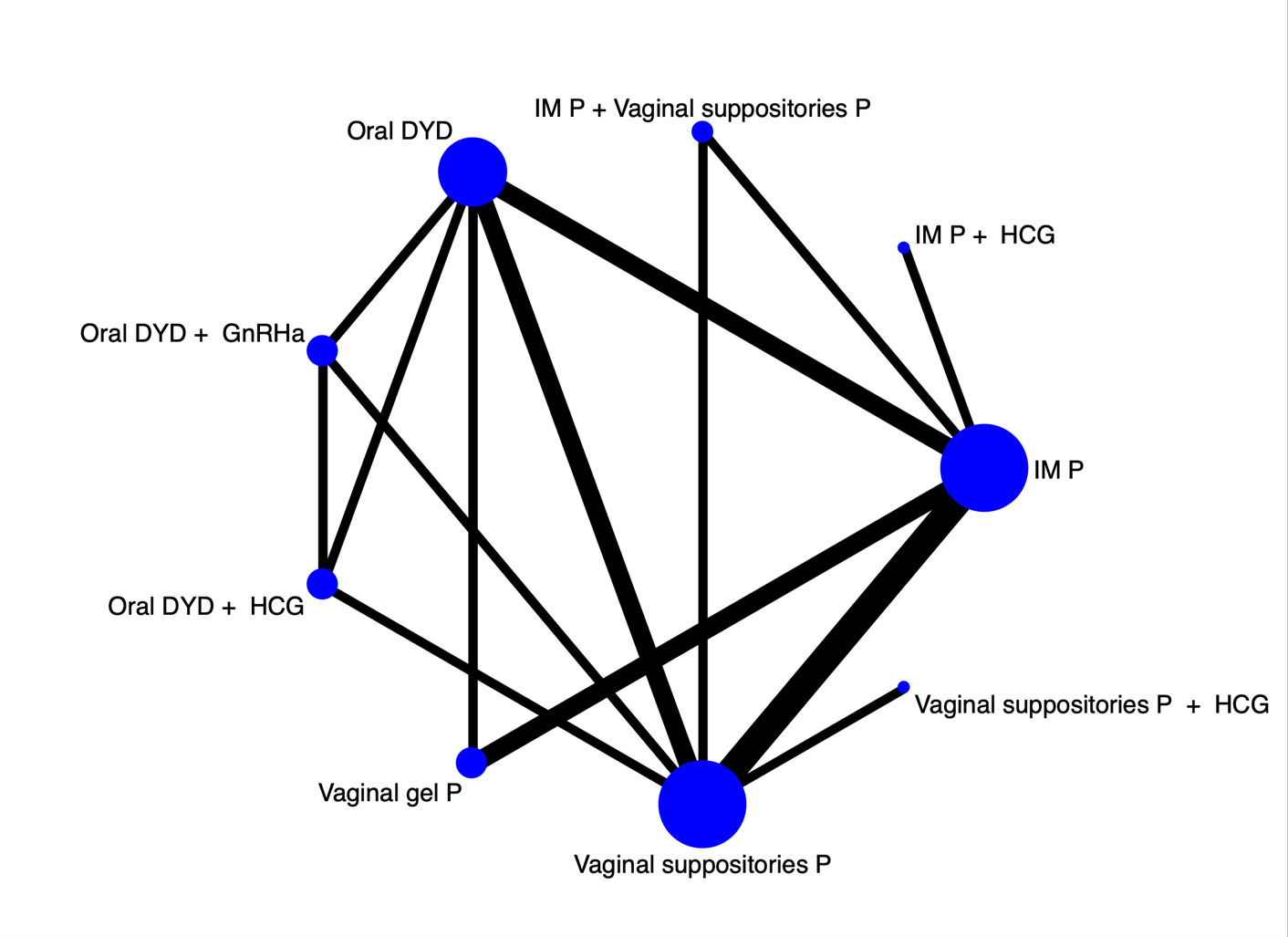
**

*Interval plot*


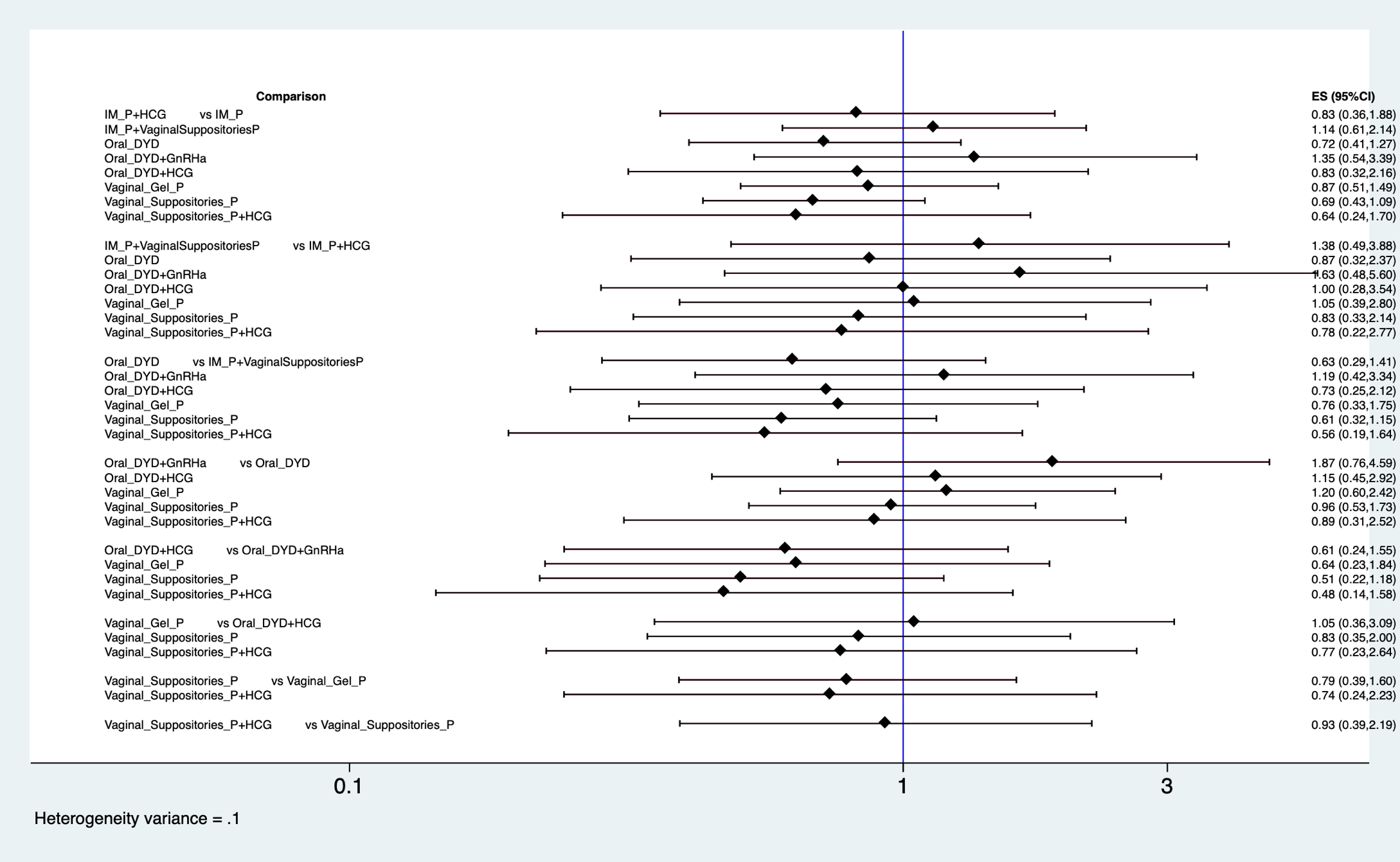


*SUCRA ranking (first three positions)*

1. Vaginal suppositories P + HCG (SUCRA = 37.6%)
2. IM P + HCG (SUCRA = 16.0%)
3. Oral DYD + HCG (SUCRA = 14.8%)
   - OPR/LBR

*Network map*


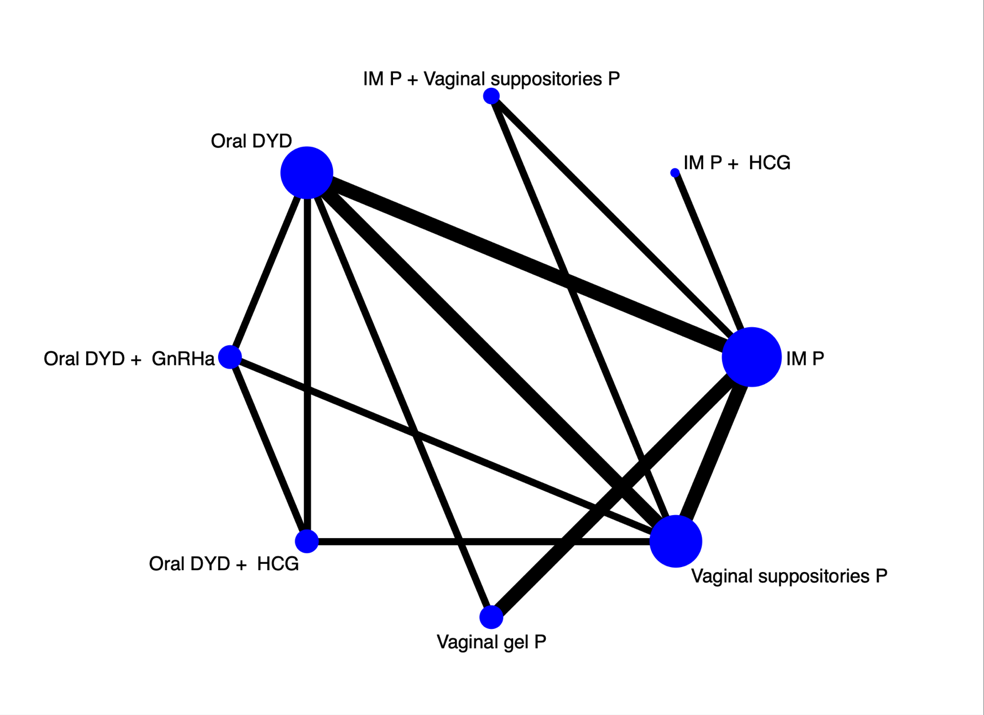


*Interval plot*


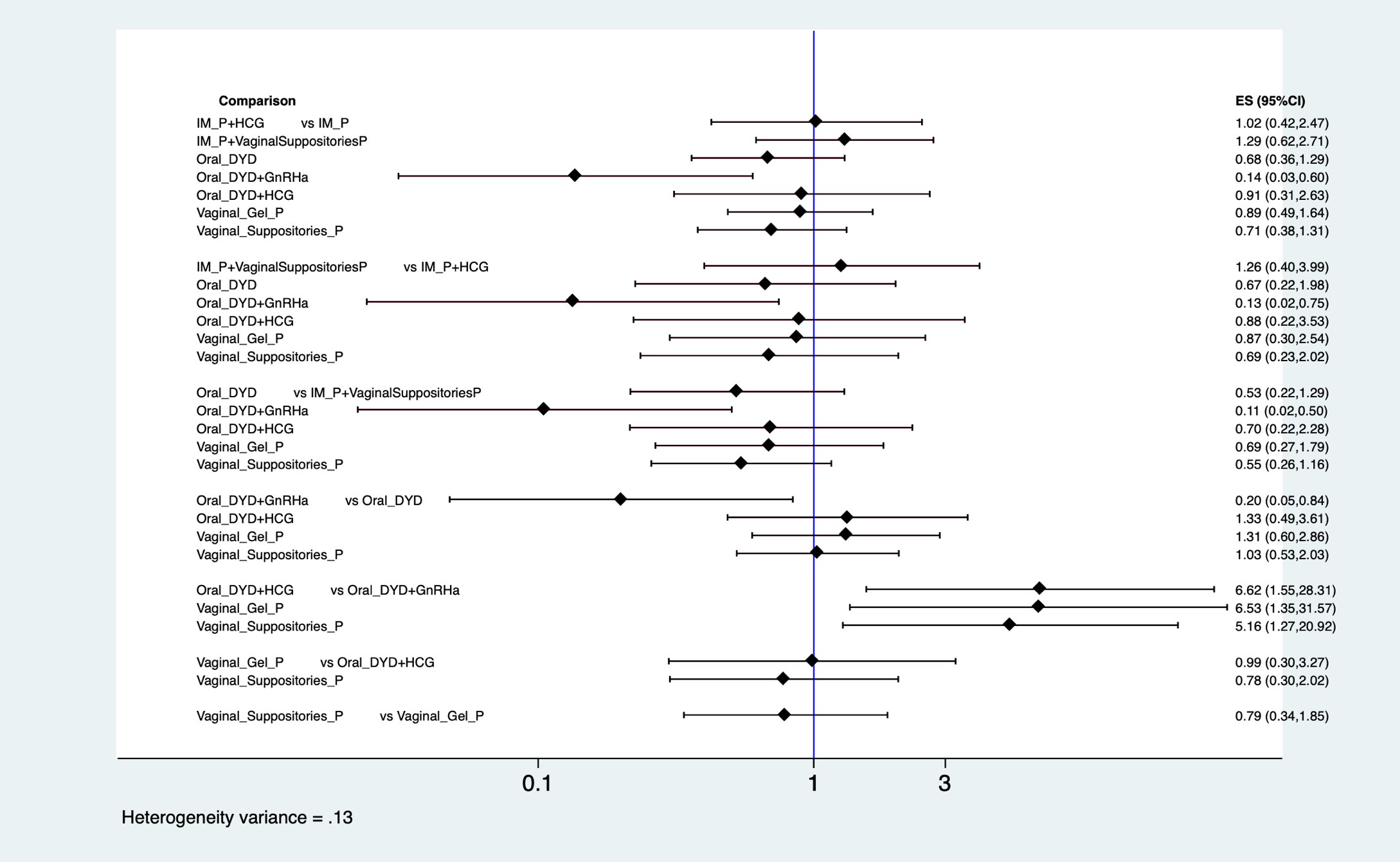


*SUCRA ranking (first three positions)*

1. Oral DYD + GnRHa (SUCRA = 97.1%)
2. IM P + HCG (SUCRA = 0.8%)
3. Vaginal suppositories P (SUCRA = 0.4%)
4. **Sensitivity analysis excluding studies with cleavage stage ET.**
   - CPR

*Network map*

*
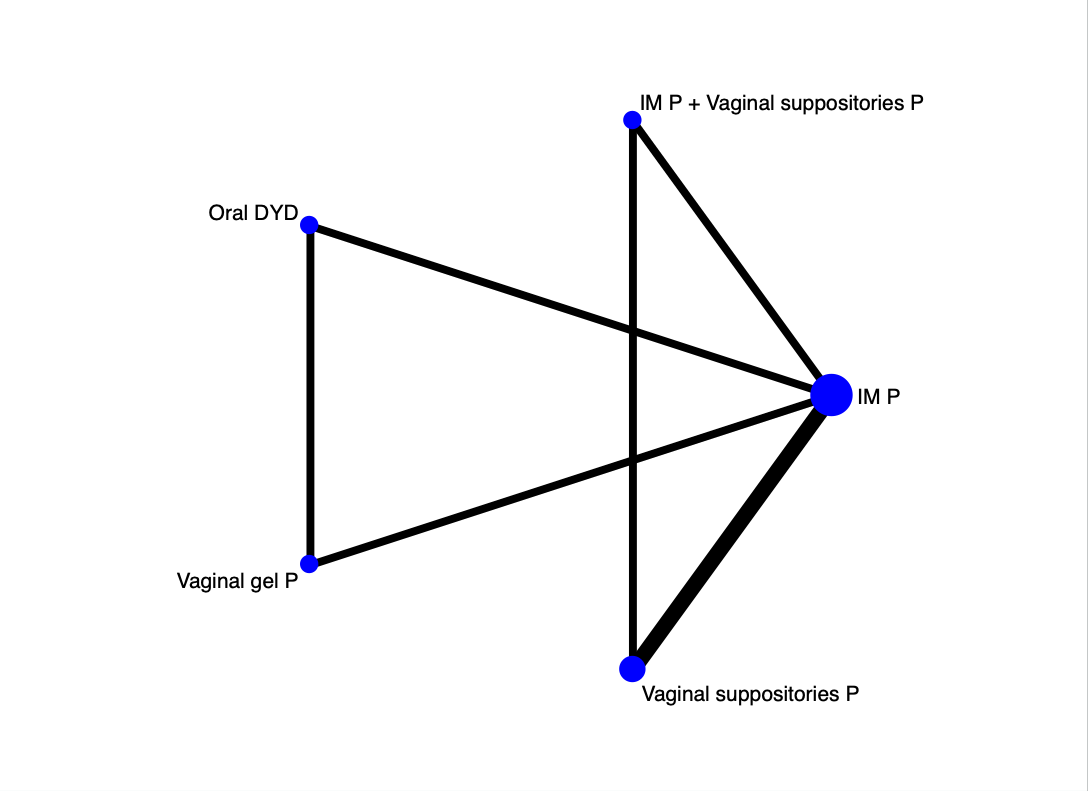
*

*Interval plot*


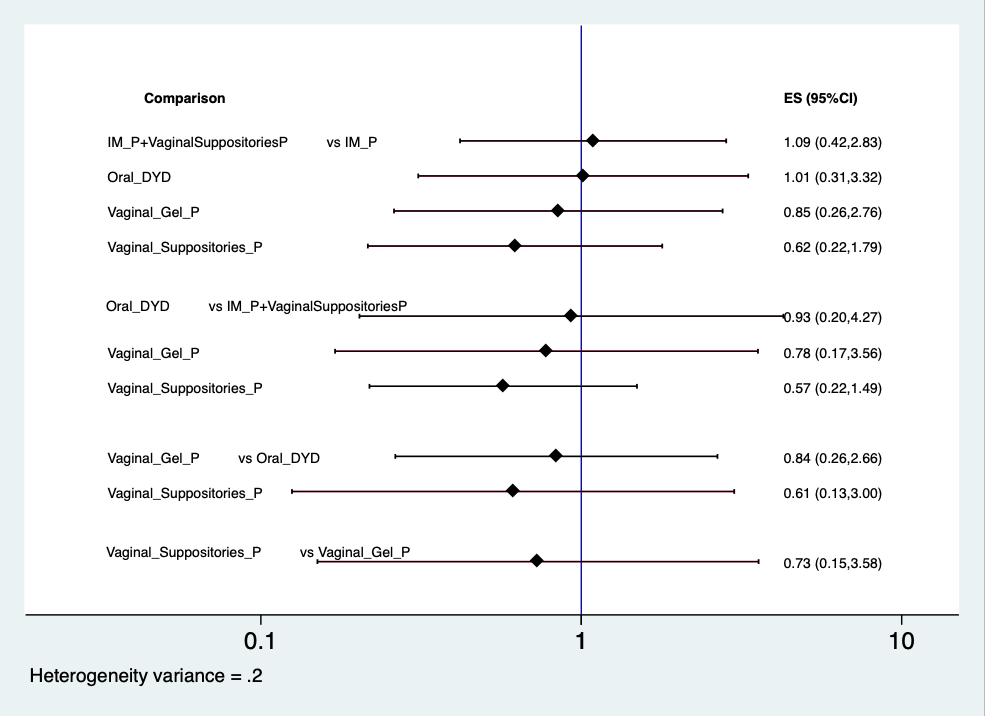


*SUCRA ranking (first three positions)*

1. Vaginal suppositories P (SUCRA = 51.9%)
2. Vaginal gel P (SUCRA = 23.9%)
3. Oral DYD (SUCRA = 14.3%)
4. IM P + Vaginal suppositories P (SUCRA = 5.2%)
5. IM P (SUCRA = 4.7%)
   - LBR

*Network map*

*
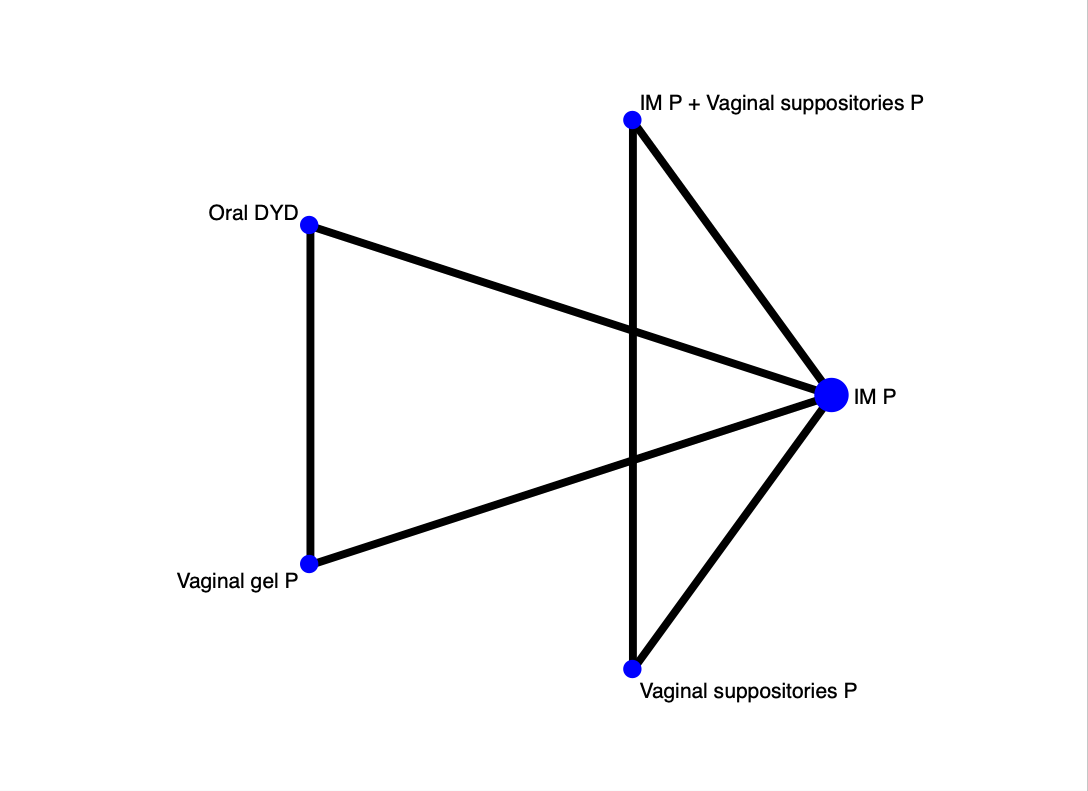
*

*Interval plot*


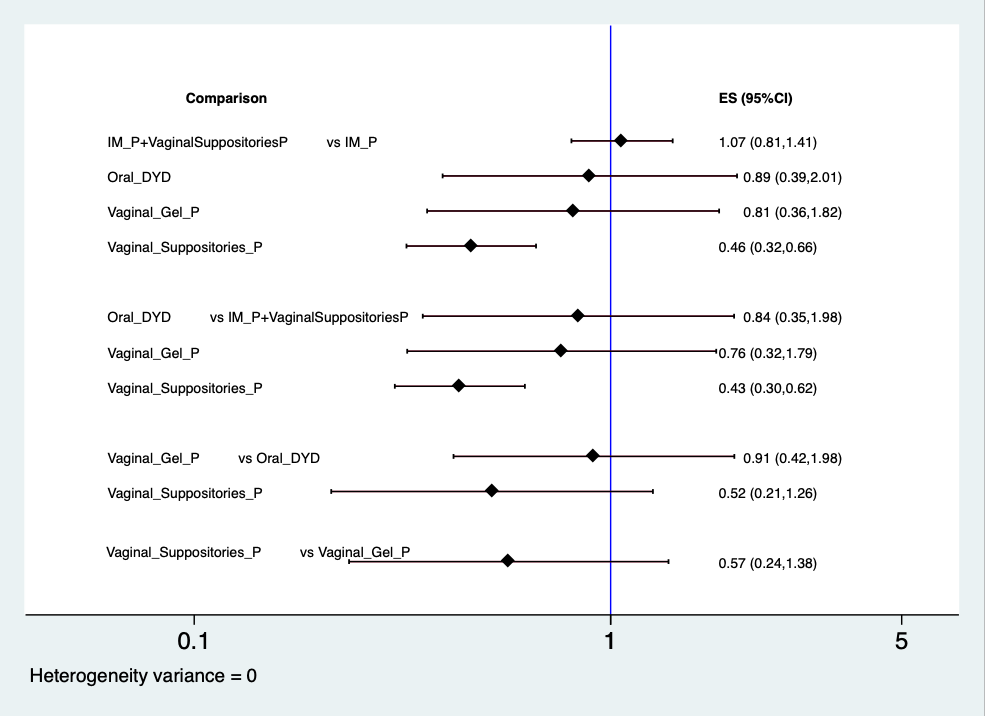


*SUCRA ranking (first three positions)*

1. Vaginal suppositories P (SUCRA = 85.7%)
2. Vaginal gel P (SUCRA = 8.9%)
3. Oral DYD (SUCRA = 5.4%)
4. **Sensitivity analysis excluding studies not reporting endometrial thickness.**
   1. CPR

*Network map*


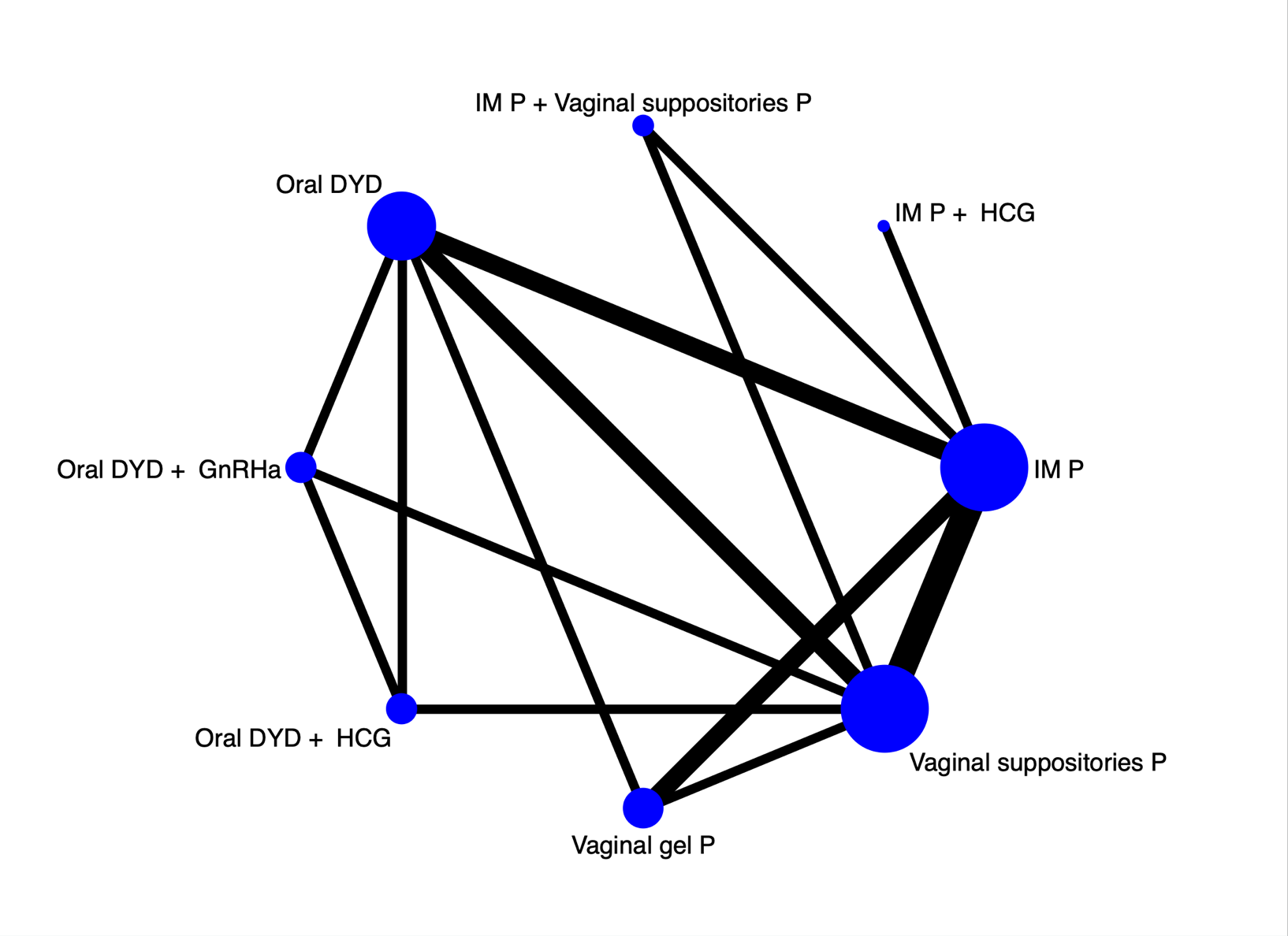


*Interval plot*


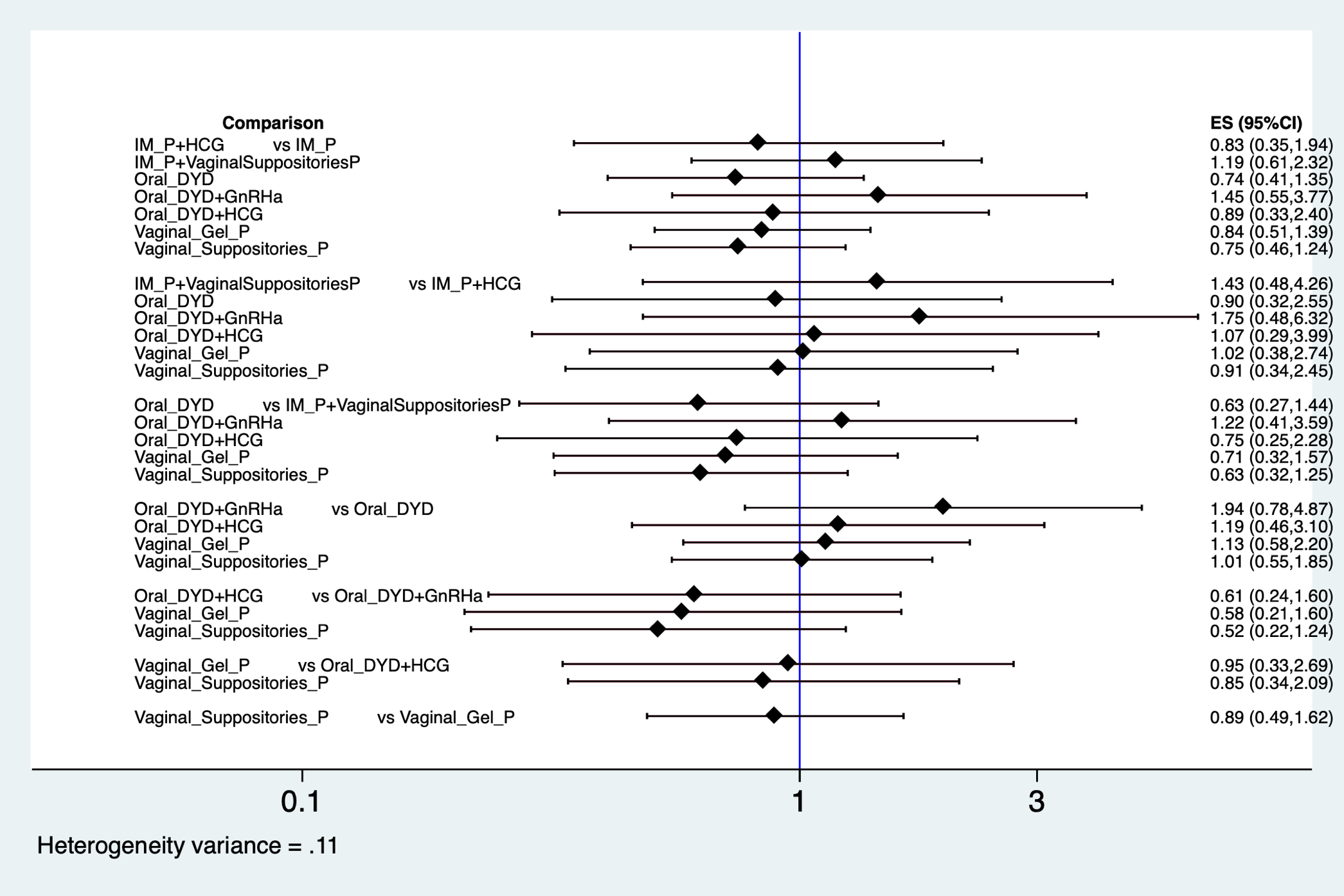


*SUCRA ranking (first three positions)*

1. IM P + HCG (SUCRA = 26.6%)
2. Oral DYD (SUCRA = 22.6%)
3. Oral DYD + HCG (SUCRA = 19.6%)
   1. OPR/LBR

Network map


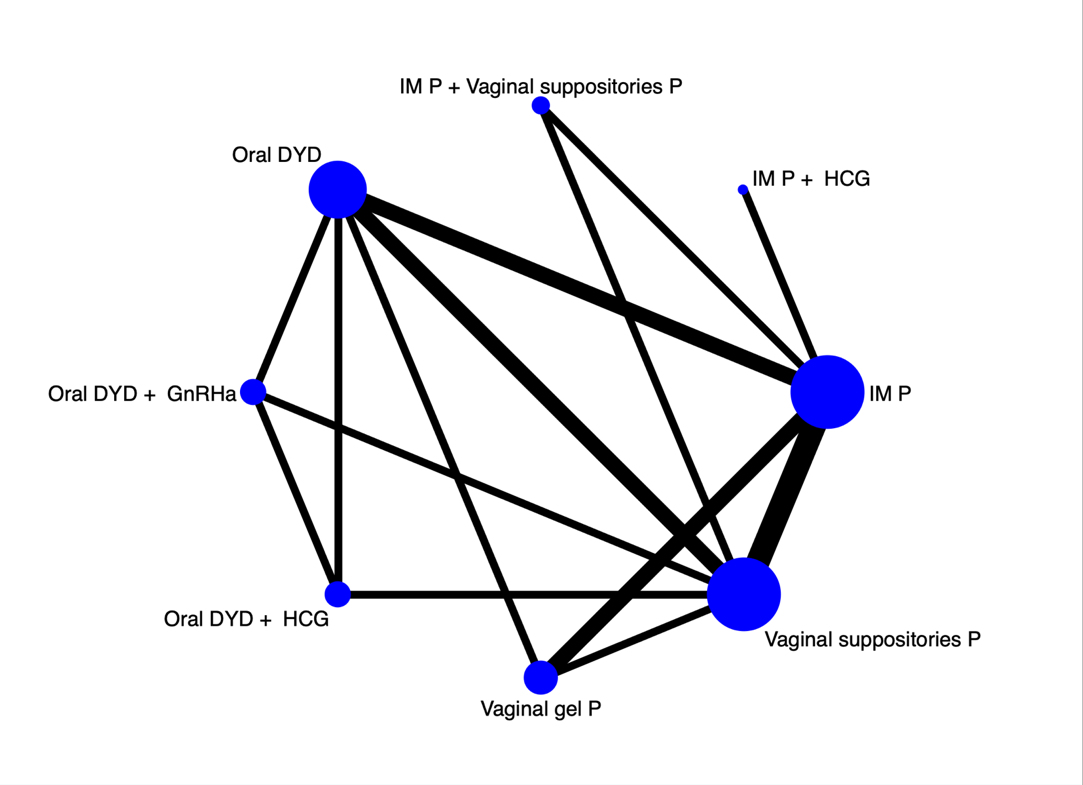


*Interval plot*


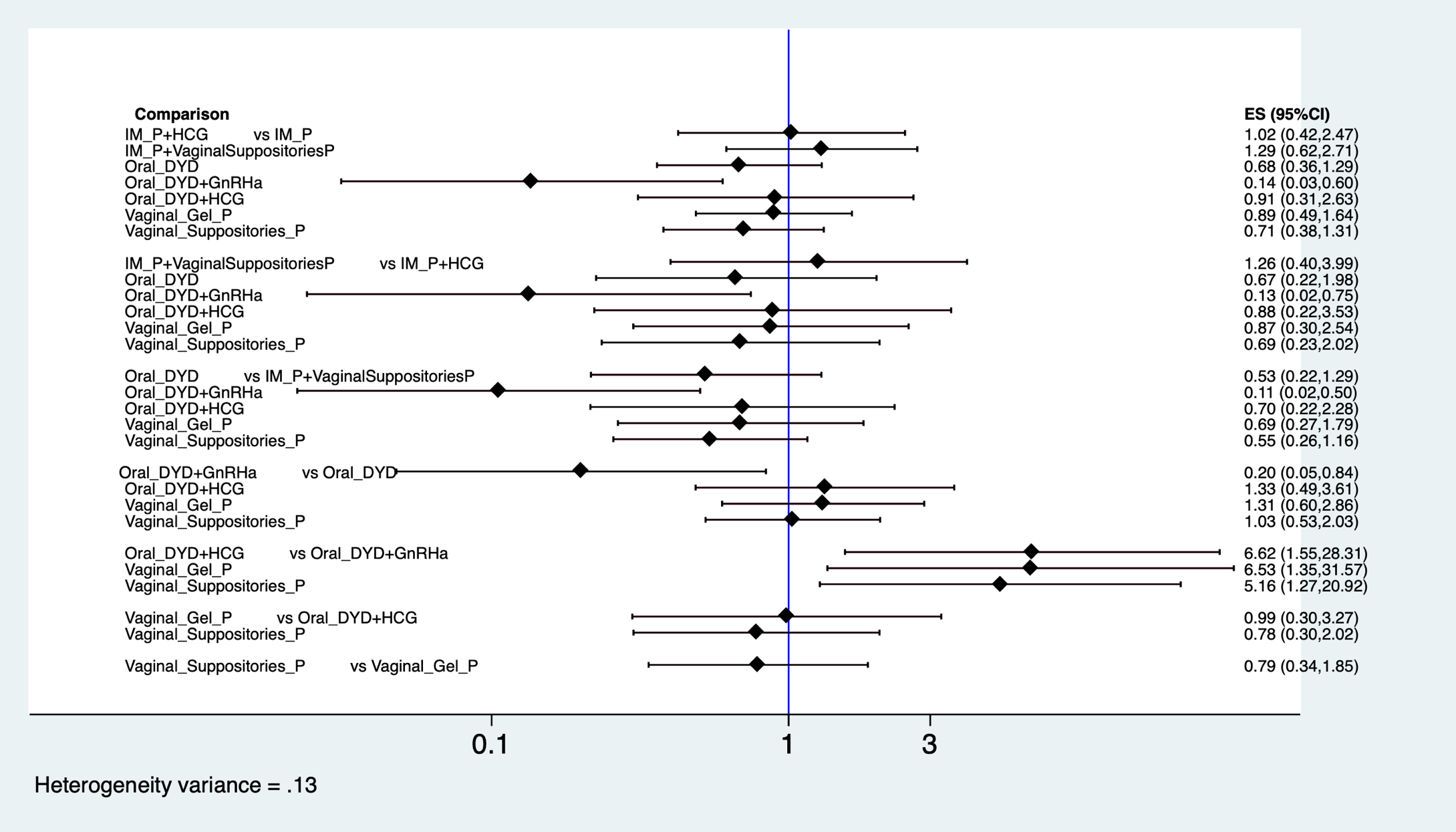


*SUCRA ranking (first three positions)*

1. Oral DYD + GnRHa (SUCRA = 97.3%)
2. Oral DYD (SUCRA = 0.8%)
3. IM P + HCG (SUCRA = 0.8%)
