# Supplementary figures and images for: Reproductive outcome after frozen embryo transfer with hormone replacement therapy according to luteal‐phase support protocol: systematic review and network meta‐analysis of randomized controlled trials
Source: Ultrasound Obstet Gynecol. 2025 Aug 1;66(4):422–32. doi: 10.1002/uog.29302 (PMC12488206; doi:10.1002/uog.29302)

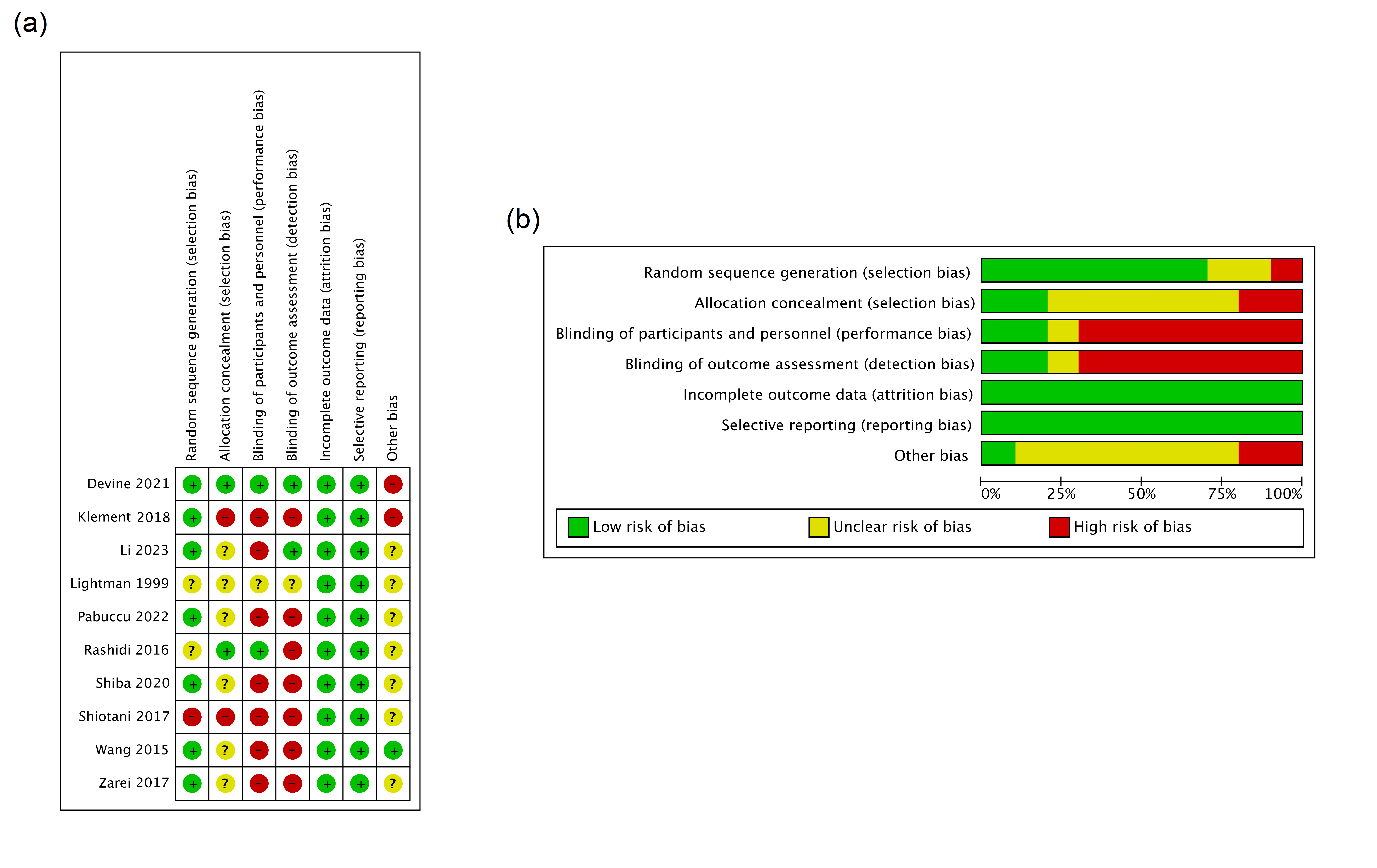

Supplement: Supplementary file 5 — Figure S1 Risk‐of‐bias assessment for included studies. Results are shown for each individual study (a) and according to each risk‐of‐bias item (b). +, low risk of bias; −, high risk of bias; ?, unclear risk of bias. [file UOG-66-422-s004.tif]

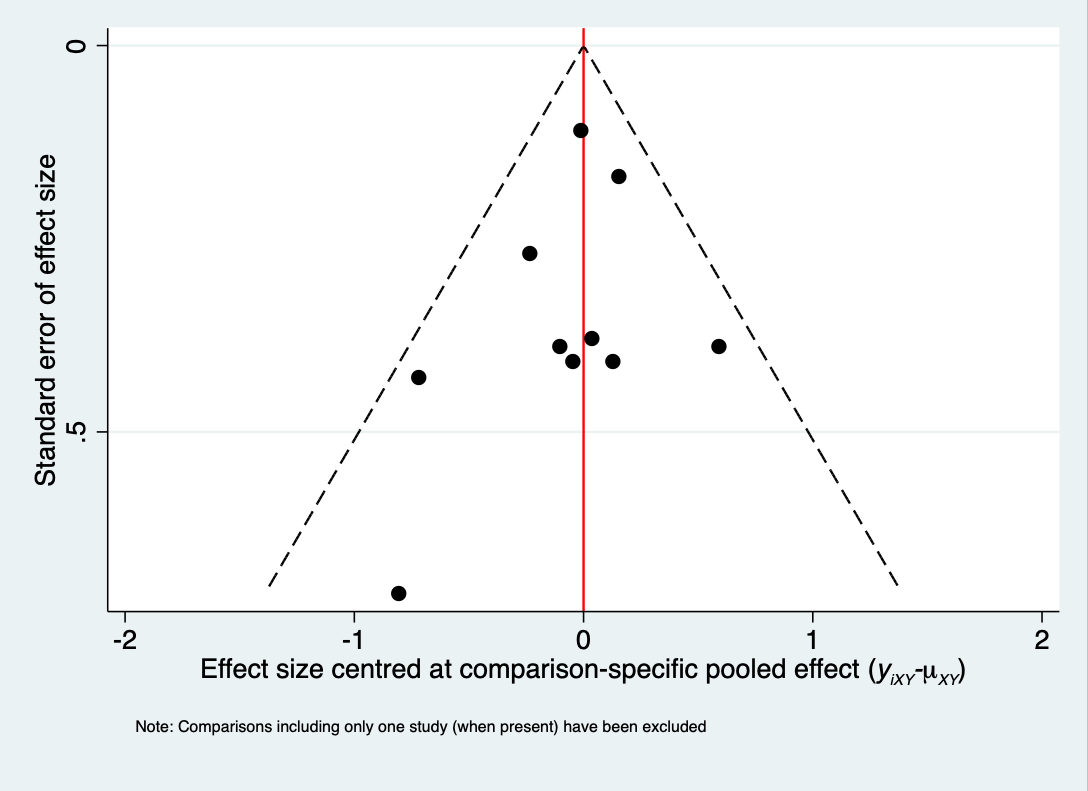

Supplement: Supplementary file 6 — Figure S2 Funnel plot for clinical pregnancy rate. [file UOG-66-422-s003.tif]
