# Supplementary material for: Reproductive outcome after frozen embryo transfer with hormone replacement therapy according to luteal‐phase support protocol: systematic review and network meta‐analysis of randomized controlled trials
Source: Ultrasound Obstet Gynecol. 2025 Aug 1;66(4):422–32. doi: 10.1002/uog.29302 (PMC12488206; doi:10.1002/uog.29302)
Supplement: Supplementary file 7 — Table S1 Description of Cochrane risk‐of‐bias tool version 1 (RoB 1) and results of quality assessment of included studies using RoB 1 [file UOG-66-422-s007.docx]

**Table S1** Description of Cochrane risk of bias (RoB) tool version 1 and results of quality assessment of included studies using RoB 1 tool

| *Reason* | *Risk of bias* | **Domain** |
| --- | --- | --- |
| Adequate randomization method described (e.g., computer-generated sequence, random number table) | Low risk | Random sequence generation (selection bias) |
| Non-random method used (e.g., alternate assignment, date of birth, open list) | High risk |  |
| Randomization method not described sufficiently | Unclear |  |
| Allocation sequence concealed until assignment (e.g., sealed opaque envelopes, central randomization) | Low risk | Allocation concealment (selection bias) |
| Allocation sequence known in advance, allowing for manipulation | High risk |  |
| No details on allocation concealment provided | Unclear |  |
| Both participants and personnel blinded; no risk of performance bias | Low risk | Blinding of participants and personnel (performance bias) |
| Participants or personnel aware of group assignment, introducing performance bias | High risk |  |
| Blinding status not reported or unclear | Unclear |  |
| Outcome assessors blinded, ensuring unbiased measurement | Low risk | Blinding of outcome assessment (detection bias) |
| Outcome assessors aware of group assignment, risking detection bias | High risk |  |
| Blinding of outcome assessment not described | Unclear |  |
| No missing data or missingness unlikely to impact results | Low risk | Incomplete outcome data (attrition bias) |
| High attrition or missing data that could influence findings, with no appropriate handling | High risk |  |
| Extent of missing data unclear or not adequately addressed | Unclear |  |
| Study protocol available and reported outcomes match pre-specified outcomes | Low risk | Selective reporting (reporting bias) |
| Selective reporting detected (e.g., important outcomes omitted, results inconsistent with trial registry) | High risk |  |
| No trial registry or unclear if all outcomes were reported | Unclear |  |
| No evidence of other biases (e.g., industry funding without transparency, imbalance in baseline characteristics) | Low risk | Other bias |
| Potential biases present (e.g., financial conflicts, baseline imbalances not adjusted for) | High risk |  |
| Unclear risk of bias due to incomplete information | Unclear |  |
|  |  | **Study** |
| *Reason/”Quotation”* | *Risk of bias* | **Devine 2021** |
| Sequential lists of randomized treatment assignments were generated by the method of randomly permuted blocks with the use of an internet-based randomization program” | Low risk | Random sequence generation (selection bias) |
| “…1:1:1 ratio. The treatment assignment  was revealed to the study coordinator at this time by opening a sequentially numbered sealed opaque envelope that contained the randomized treatment assignment.” | Low risk | Allocation concealment (selection bias) |
| “The study team, including the statistician, were blinded to the identity of the treatment arms” | Low risk | Blinding of participants and personnel (performance bias) |
| “…so that the analysis of any potential group differences could be conducted in a blinded fashion” | Low risk | Blinding of outcome assessment (detection bias) |
| Low rate of patients lost during follow-up | Low risk | Incomplete outcome data (attrition bias) |
| Protocol publicly available and respected | Low risk | Selective reporting (reporting bias) |
|  | High risk | Other bias |
| *Reason/”Quotation”* | *Risk of bias* | **Klement 2018** |
| Randomization method only partly described | Unclear risk | Random sequence generation (selection bias) |
| Not enough details regarding allocation sequence | High risk | Allocation concealment (selection bias) |
| “This was a randomized non-blinded study…” | High risk | Blinding of participants and personnel (performance bias) |
| “This was a randomized non-blinded study…” | High risk | Blinding of outcome assessment (detection bias) |
| Non-significant loss of patients during follow-up | Low risk | Incomplete outcome data (attrition bias) |
| Study protocol available and reported outcomes match pre-specified outcomes | Low risk | Selective reporting (reporting bias) |
| Extremely scarce reporting of baseline data | High risk | Other bias |
| *Reason/”Quotation”* | *Risk of bias* | **Li 2023** |
| “Women were randomly allocated to the hCG treatment or the control group according to a randomization list generated by a computer. The specific process was as follows: 300 random numbers were produced by a computer and divided into A and B groups, with 150 in each group.” | Low risk | Random sequence generation (selection bias) |
| “Then a random group table was made and blinded on computer. The 300 random numbers obtained above were randomly distributed to 300 sequence numbers. For every patient included, a random number was obtained according to the order of inclusion. Then the number was unblinded by the computer” | Unclear | Allocation concealment (selection bias) |
| “…the staff who conduct the randomization process and the participants were not blinded” | High risk | Blinding of participants and personnel (performance bias) |
| “…Laboratory staff and staff who conducted the data analysis and follow-up were blinded...” | Low risk | Blinding of outcome assessment (detection bias) |
| Zero losses at follow-up | Low risk | Incomplete outcome data (attrition bias) |
| Protocol publicly available and respected | Low risk | Selective reporting (reporting bias) |
| Only few baseline characteristics reported | Unclear | Other bias |
| *Reason/”Quotation”* | *Risk of bias* | **Lightman 1999** |
| Randomization method not described sufficiently | Unclear | Random sequence generation (selection bias) |
| Allocation made by last digits of patient ID without additional information | Unclear | Allocation concealment (selection bias) |
| Personnel and participants were both blinded | Unclear | Blinding of participants and personnel (performance bias) |
| Blinding status not reported | Unclear | Blinding of outcome assessment (detection bias) |
| No attrition bias concerns | Low risk | Incomplete outcome data (attrition bias) |
| All the outcomes reported. Protocol registration not required (enrollment prior to 2010) | Low risk | Selective reporting (reporting bias) |
| Incomplete baseline information | Unclear | Other bias |
| *Reason/”Quotation”* | *Risk of bias* | **Pabuccu 2022** |
| “were randomly assigned based on a computer-generated list…” | Low risk | Random sequence generation (selection bias) |
| No details on allocation concealment provided | Unclear | Allocation concealment (selection bias) |
| “The study was not blinded (open-  label) as it was deemed technically not feasible to make placebo arrangements for all three regimens” | High risk | Blinding of participants and personnel (performance bias) |
| “The study was not blinded (open-  label) as it was deemed technically not feasible to make placebo arrangements for all three regimens” | High risk | Blinding of outcome assessment (detection bias) |
| Zero losses at follow-up | Low risk | Incomplete outcome data (attrition bias) |
| All the outcomes reported. Protocol registration provided. | Low risk | Selective reporting (reporting bias) |
| Only few baseline characteristics reported | Unclear | Other bias |
| *Reason/”Quotation”* | *Risk of bias* | **Rashidi 2016** |
| Randomization method not described sufficiently | Unclear | Random sequence generation (selection bias) |
| Allocation made by last digits of patient ID without additional information | Low risk | Allocation concealment (selection bias) |
| “Single blinding was done by keeping the person enrolling the participants and the study investigators uninformed of the type of the treatment protocol” | Low risk | Blinding of participants and personnel (performance bias) |
| Personnel evaluating the outcomes was unblinded. | High risk | Blinding of outcome assessment (detection bias) |
| No attrition bias concerns | Low risk | Incomplete outcome data (attrition bias) |
| All the outcomes reported. Protocol registration provided. | Low risk | Selective reporting (reporting bias) |
| Incomplete baseline information reported | Unclear | Other bias |
| *Reason/”Quotation”* | *Risk of bias* | **Shiba 2020** |
| “…a specific researcher generated a computer‐based random allocation table, and all patients were randomly assigned to one of the four study groups” | Low risk | Random sequence generation (selection bias) |
| Allocation concealment not reported | Unclear | Allocation concealment (selection bias) |
| Personnel and participants were both unblinded. | High risk | Blinding of participants and personnel (performance bias) |
| Outcome assessor was unblinded. | High risk | Blinding of outcome assessment (detection bias) |
| No attrition bias concerns | Low risk | Incomplete outcome data (attrition bias) |
| All the outcomes reported. Protocol registration provided. | Low risk | Selective reporting (reporting bias) |
| Unclear baseline data regarding ovarian reserve | Unclear | Other bias |
| *Reason/”Quotation”* | *Risk of bias* | **Shiotani 2017** |
| Randomization method not mentioned | High risk | Random sequence generation (selection bias) |
| Allocation methodology not clearly distinguishable | High risk | Allocation concealment (selection bias) |
| Personnel and participants might have been aware of group assignment | High risk | Blinding of participants and personnel (performance bias) |
| Personnel in charge of outcome assessment might have been aware of group division | High risk | Blinding of outcome assessment (detection bias) |
| No attrition bias concerns | Low risk | Incomplete outcome data (attrition bias) |
| All the outcomes reported. Protocol registration not required (enrollment prior to 2010) | Low risk | Selective reporting (reporting bias) |
| Unclear due to incomplete baseline information | Unclear | Other bias |
| *Reason/”Quotation”* | *Risk of bias* | **Wang 2015** |
| “A computer-based random allocation table was generated by researchers who were blind to this study. The table randomized the 1,500 cycles into the two groups using 1,500 numbers… “ | Low risk | Random sequence generation (selection bias) |
| 1:1 ratio allocation but not clearly explained | Unclear | Allocation concealment (selection bias) |
| “… researchers and patients were informed of the grouping results on day 0. Masking or blinding was not possible in this study.” | High risk | Blinding of participants and personnel (performance bias) |
| “… researchers and patients were informed of the grouping results on day 0. Masking or blinding was not possible in this study.” | High risk | Blinding of outcome assessment (detection bias) |
| No attrition bias concerns | Low risk | Incomplete outcome data (attrition bias) |
| All the outcomes reported. Protocol registration provided. | Low risk | Selective reporting (reporting bias) |
| No other bias to report | Low risk | Other bias |
| *Reason/”Quotation”* | *Risk of bias* | **Zarei 2017** |
| “All the patients were given a registration number based on the order of their referral. They were randomly assigned to four study groups using a computer-based random digit generator” | Low risk | Random sequence generation (selection bias) |
| Allocation methodology not reported | Unclear | Allocation concealment (selection bias) |
| Personnel and participants might have been aware of group assignment | High risk | Blinding of participants and personnel (performance bias) |
| Personnel in charge of outcome assessment might have been aware of group division | High risk | Blinding of outcome assessment (detection bias) |
| No attrition bias concerns | Low risk | Incomplete outcome data (attrition bias) |
| All the outcomes reported. Protocol registration provided. | Low risk | Selective reporting (reporting bias) |
| Unclear due to paucity of baseline information | Unclear | Other bias |
