# Supplementary material for: Reproductive outcome after frozen embryo transfer with hormone replacement therapy according to luteal‐phase support protocol: systematic review and network meta‐analysis of randomized controlled trials
Source: Ultrasound Obstet Gynecol. 2025 Aug 1;66(4):422–32. doi: 10.1002/uog.29302 (PMC12488206; doi:10.1002/uog.29302)
Supplement: Supplementary file 8 — Table S2 Characteristics of studies included in quantitative analysis [file UOG-66-422-s008.docx]

**Table S2** Characteristics of studies included in quantitative analysis

| **Author** | **Year of publication** | **Type** | **Main outcomes** | **Treatment** | **Trial registration** | **Inclusion criteria** | **Exclusion criteria** |
| --- | --- | --- | --- | --- | --- | --- | --- |
| Lightman et al. | 1999 | RCT | Clinical pregnancy rate and abortion rate | - Group 1: Vaginal suppositories P - Group 2: IM P | Not available (recruitment started more than 15 years ago) | - Infertile women underwent HRT-FET | - No luteal phase support (i.e. natural cycle). |
| Wang et al. | 2015 | RCT | Live birth rate, clinical pregnancy rate, abortion rate and ectopic pregnancy rate | - Group 1: Vaginal gel P - Group 2: IM P | ChiCTR-TRC-14004565 | - Patients aged between 20 and 40 - Day 3 frozen embryos - endometrial thickness >=7 mm on the secretory transformation day. | - Patient with uterine disorders, adenomyosis, submucous myoma or intrauterine adhesions - Patients with a history of natural abortions (including biochemical pregnancies) or embryo transplant failures (including biochemical pregnancies) on more than three occasions - Patients taking drugs or therapies that may affect reproductive or metabolic functions, such as anti-diabetic drugs, anti-hypertensive drugs (including diazoxide, ACE inhibitors and calcium channel blockers), Chinese herbal medicines and acupuncture - Patients with endometrial thickness <7 mm on the secretory transformation day - Patients who were unable to comply with the study protocol |
| Rashidi et al. | 2016 | RCT | Clinical pregnancy rate, ongoing pregnancy rate, miscarriage rate | - Group 1: Vaginal suppositories P - Group 2: Oral DYD - IM P | IRCT201406255181N15 | - Patients undergoing FET, because of leftover embryos from past fresh or frozen cycles, canceled previous cycles, because of bad endometrium or ovarian hyper stimulation syndrome or candidates for embryo donation | - Women with other indications and 64 methods of ART |
| Zarei et al. | 2017 | RCT | Clinical pregnancy rate, ongoing pregnancy rate, miscarriage rate | - Group 1: Vaginal suppositories P - Group 2: Oral DYD - Group 3: Oral DYD + GnRHa - Group 4: Oral DYD+hCG | IRCT2014082510210N3 | - Patients with unexplained infertility, tubal factor infertility, mild male factor infertility, premature ovarian failure (POF), polycystic ovarian syndrome (PCOS), endometriosis stage I or II, and normal uterine cavity, who were 20–40 years | - Older than 40 years - endometriosis stage III or IV - hydrosalpinx - severe male factor infertility - abnormal uterine cavity - three or more unsuccessful embryo transfer cycles - less than two top quality embryos in women below than 35 years of age - less than three top quality embryos in those between 35 and 40 years of age |
| Shiotani et al. | 2017 | RCT | Clinical pregnancy rate, miscarriage rate | - Group 1: Vaginal suppositories P + hCG - Group 2: Vaginal suppositories P | Not available (recruitment started more than 15 years ago) | - Not specified | - Not specified |
| Klement et al. | 2018 | RCT | Clinical pregnancy rate | - Group 1: Vaginal suppositories P - Group 2: IM P | NCT02078869 | - Patients assigned to a blastocyst FET cycle, either using autolo- gous eggs or fertilized donor eggs | - Patients with uterine factor infertility - Patients previously treated by P LPS and who had a known adverse effect related to the treatment or the treatment modality. |
| Devine et al. | 2021 | RCT | Ongoing pregnancy rate | - Group 1: IM P - Group 2: Vaginal suppositories P - Group 3: Vaginal suppositores P + IM P | NCT02254577 | - female age 18–48 years; - Having available blastocyst(s) cryopreserved by vitrification method at SGF | - requirement for fresh embryo(s); - requirement for a gestational carrier; - embryo(s) for transfer from cryopreserved oocytes; - embryo(s) for transfer cryopreserved more than once; - embryo(s) for transfer cryopreserved by slow-freeze method; - embryo(s) for transfer cryopreserved before blastocyst stage; - presence of any clinically relevant systemic disease contraindicated for assisted reproduction or pregnancy; - history of more than three failed cycles of assisted reproduction or more than three clinical pregnancy losses after embryo transfer; - surgical or medical condition or requirement for medication that may interfere with absorption, distribution, metabolism, or excretion of the drugs to be used; - body mass index of <18 or >38 kg/m2 at screening; - current or recent (within 3 months) substance abuse, including alcohol and tobacco; - current breastfeeding or pregnancy; - refusal or inability to comply with the requirements of the protocol for any reason, including scheduled clinic visits and laboratory tests; - trophectoderm or blastomere biopsy of the blastocyst(s) to be transferred; - intolerance or allergy to any of the medications used in the study protocol; ( - participation in any experimental drug study within 60 days before screening; - two previous study cycles (subjects were permitted to participate twice). |
| Shiba et al. | 2020 | RCT | Clinical pregnancy rate and miscarriage rate | - Group 1: Vaginal suppositories P - Group 2: Vaginal gel P | UMIN000032997 | - Patients who underwent egg retrieval, and had at least one embryo frozen using the vitrification method for cryopreservation | - Patients who had contraindications that were described on the drug package - Patients who desired to transfer two embryos - Patients who desired the use of a different preparation method |
| Pabuccu et al. | 2022 | RCT | Live birth rate, ongoing pregnancy rate, biochemical pregnancy rate, clinical miscarriages and side effects. | - Group 1: Oral DYD - Group 2: Vaginal gel P - Group 3: IM P | NCT03948022 | - women meeting the requirement of IVF or treatment - age ≤40 years at the time of egg retrieval - patients enrolled in an FET cycle due to an excessive ovarian response or other indications - programmed FET cycles using oral estradiol - women with at least one autologous cryopreserved blastocyst endometrial thickness ≥7 mm and serum progesterone <1.5 ng/ml before the start of progesterone treatment. | - ≥3 previous unsuccessful IVF cycles and ≥2 early pregnancy losses - any uterine anomaly documented by hysterosalpingography or 3D sonography - a requirement for fresh embryo(s) - the presence of any clinically relevant systemic disease contraindicating assisted reproduction or pregnancy - a history of allergy for the relevant drugs - a body mass index of <18 or >38 kg/m at screening - current breastfeeding or pregnancy refusal or an inability to comply for any reason with the requirements of the protocol, including scheduled clinic visits and laboratory tests - trophectoderm or blastomere biopsy of the blastocysts) to be transferred - women enrolled for natural or modified natural cycle FET. |
| Li et al. | 2023 | RCT | Live birth rate, clinical pregnancy, miscarriage rate and neonatal outcomes | - Group 1: IM P + hCG - Group 2: IM P | ChiCTR1800020342 | - age < 40 years - first FET cycle - artificial cycle for endometrium preparation | - Women with confirmed endometriosis, uterine malformation, intrauterine adhesion or untreated hydrosalpinx - Cycles were not eligible if the endometrial thickness was ≤ 8 mm before starting progesterone. - Cycles with follicular diameter >14 mm on the day of progesterone administration were also excluded. - Women participating in other studies. |

RCT: randomized controlled trial; FET: frozen embryo transfer; NC: natural cycle; AC: artificial cycle; HRT: hormone replacement therapy; LBR: live birth rate; CPR: clinical pregnancy rate; MR: miscarriage rate; OPR: ongoing pregnancy rate; OR: odd ratio; CI: confidence interval; LPS: luteal phase support; IM: intramuscular; SC: subcutaneous; hCG: human chorionic Gonadotropin; P: progesterone; GnRHa: gonadotropin Releasing hormone agonist; IVF: in vitru fertilization; ART: assisted reproductive technology; PGT: preimplantation genetic testing.

**References**

Lightman A, Kol S, Itskovitz-Eldor J. A prospective randomized study comparing intramuscular with intravaginal natural progesterone in programmed thaw cycles. *Hum Reprod*. 1999;14(10):2596-2599.

Wang Y, He Y, Zhao X, et al. Crinone Gel for Luteal Phase Support in Frozen-Thawed Embryo Transfer Cycles: A Prospective Randomized Clinical Trial in the Chinese Population. *PLoS One*. 2015;10(7):e0133027.

Rashidi BH, Ghazizadeh M, Tehrani Nejad ES, Bagheri M, Gorginzadeh M. Oral dydrogesterone for luteal support in frozen-thawed embryo transfer artificial cycles: A pilot randomized controlled trial. *Asian Pacific Journal of Reproduction*. 2016;5(6):490-494.

Zarei A, Sohail P, Parsanezhad ME, Alborzi S, Samsami A, Azizi M. Comparison of four protocols for luteal phase support in frozen-thawed Embryo transfer cycles: a randomized clinical trial. *Arch Gynecol Obstet*. 2017;295(1):239-246.

Shiotani M, Matsumoto Y, Okamoto E, et al. Is human chorionic gonadotropin supplementation beneficial for frozen and thawed embryo transfer in estrogen/progesterone replacement cycles?: A randomized clinical trial. *Reprod Med Biol*. 2017;16(2):166-169.

Klement AH, Samara N, Weintraub A, et al. Intramuscular versus Vaginal Progesterone Administration in Medicated Frozen Embryo Transfer Cycles: A Randomized Clinical Trial Assessing Sub-Endometrial Contractions. *Gynecol Obstet Invest*. 2018;83(1):40-44.

Devine K, Richter KS, Jahandideh S, Widra EA, McKeeby JL. Intramuscular progesterone optimizes live birth from programmed frozen embryo transfer: a randomized clinical trial. *Fertil Steril*. 2021;116(3):633-643.

Shiba R, Kinutani M, Okano S, Kawano R, Kikkawa Y. Efficacy of four vaginal progesterones for luteal phase support in frozen-thawed embryo transfer cycles: A randomized clinical trial. *Reprod Med Biol*. 2020;19(1):42-49.

Pabuccu E, Kovanci E, Israfilova G, Tulek F, Demirel C, Pabuccu R. Oral, vaginal or intramuscular progesterone in programmed frozen embryo transfer cycles: a pilot randomized controlled trial. *Reprod Biomed Online*. 2022;45(6):1145-1151.

Li X, Huang Y, Shi Z, Shi J, Li N. Intramuscular injection of human chorionic gonadotropin as luteal phase support in artificial cycle frozen-thawed embryo transfer does not improve clinical outcomes: a parallel, open-label randomized trial. *Front Endocrinol (Lausanne)*. 2023;14:1283197.
