# Supplementary material for: Reproductive outcome after frozen embryo transfer with hormone replacement therapy according to luteal‐phase support protocol: systematic review and network meta‐analysis of randomized controlled trials
Source: Ultrasound Obstet Gynecol. 2025 Aug 1;66(4):422–32. doi: 10.1002/uog.29302 (PMC12488206; doi:10.1002/uog.29302)
Supplement: Supplementary file 9 — Table S3 Evaluation of local inconsistency for study outcomes using separating indirect from direct evidence (SIDE)‐splitting analysis [file UOG-66-422-s002.docx]

**Table S3** Evaluation of local inconsistency for study outcomes using separating indirect from direct evidence (SIDE)-splitting analysis.

***OPR/LBR***

no source of heterogeneity. SIDE-analysis not required.

***CPR***

Side Direct Indirect Difference

Coef. Std. Err. Coef. Std. Err. Coef. Std. Err. P>|z|

A B . . . . . . .

A C * -.0285091 .143935 1.150143 .4670267 -1.178652 .4881929 0.016

A D -.0250054 .3273299 -.8191611 .4454335 .7941557 .5548773 0.152

A G -.0629905 .2490355 -.4918866 .4491942 .4288961 .5182707 0.408

A H * -.5168607 .1825855 .2317233 .3338545 -.7485839 .363473 0.039

C H * -.6845195 .174372 .4941412 .4347976 -1.178661 .4881928 0.016

D E * 1.215023 .4378127 -1.508444 .8868043 2.723467 1.056236 0.010

D F * .7280077 .4574034 -1.99546 .8966969 2.723468 1.056286 0.010

D G -.1774021 .4791973 .3585884 .4061231 -.5359905 .6278228 0.393

D H * .2289158 .3348224 -.4225127 .4321995 .6514285 .5453686 0.232

E F . . . . . . .

E H * -.2876821 .3634293 -3.011146 .9825001 2.723464 1.056277 0.010

F H * .1993329 .3868068 -2.52413 .9914032 2.723463 1.056294 0.010

G H -.0226688 .4484053 -.1853664 .3573525 .1626976 .573383 0.777

H I * -.0723465 .4105531 .5994056 1476.08 -.6717521 1476.08 1.000

*: all the evidence about these contrasts comes from the trials which directly compare them.

***PLR***

Side Direct Indirect Difference

Coef. Std. Err. Coef. Std. Err. Coef. Std. Err. P>|z|

A B * -.3513649 .3105495 -1.022439 1.095022 .6710737 1.134766 0.554

A C .5061141 .7617695 .5687646 .7027711 -.0626505 1.083242 0.954

A F -.115041 .2033844 .7825687 .53403 -.8976097 .5738946 0.118

A G * .4204517 .1826926 -.3600399 .499149 .7804916 .5317278 0.142

B G * .7601733 .3233694 .0891009 1.083243 .6710723 1.134204 0.554

C D * -.2336149 .7367343 -.3015285 1.862899 .0679136 1.978229 0.973

C E * -.2336149 .7367357 -.3015229 1.863036 .0679081 1.978359 0.973

C F -.3930156 .8318762 -.4640502 .6931889 .0710346 1.085658 0.948

C G * -.3071269 .6321736 -.1663638 .799456 -.140763 1.031924 0.891

D E . . . . . . .

D G * -2.98e-12 .7698507 -.0679082 1.822333 .0679082 1.978274 0.973

E G * 7.29e-11 .7698507 -.0679082 1.822433 .0679082 1.978366 0.973

F G -.4267749 .5192435 .5247003 .2663337 -.9514752 .5835645 0.103

G H * -.1823216 .6608532 -.6263119 3025.631 .4439903 3025.631 1.000

*: all the evidence about these contrasts comes from the trials which directly compare them.
